# A Preliminary Evaluation of The Karst Flora of Brazil Using Collections Data

**DOI:** 10.1038/s41598-019-53104-6

**Published:** 2019-11-19

**Authors:** Nadia Bystriakova, Pablo Hendrigo Alves De Melo, Justin Moat, Eimear Nic Lughadha, Alexandre K. Monro

**Affiliations:** 1 Core Research Laboratories, The Natural History Museum, London, SW7 5BD UK; 2 UNESP - Universidade Estadual Paulista “Júlio de Mesquita Filho”, Av. 24-A 1515 - Bela Vista, CEP 13506-900 Rio Claro, São Paulo, SP Brazil; 3 Biodiversity Informatics and Spatial Analysis, Royal Botanic Gardens, Kew, TW9 3AE UK; 4 School of Geography, University of Nottingham, Nottingham, NG7 2RD UK; 5 Conservation Science, Royal Botanic Gardens, Kew, TW9 3AE UK; 6 Identification and Naming, Royal Botanic Gardens, Kew, TW9 3AE UK

**Keywords:** Statistical methods, Biogeography, Conservation biology, Plant ecology

## Abstract

Karst is defined as landscapes that are underlain by soluble rock in which there is appreciable water movement arising from a combination of high rock solubility and well-developed secondary (fracture) porosity. Karsts occupy approximately 20% of the planet’s dry ice-free land and are of great socioeconomic importance, as they supply water to up to 25% of the world’s population and represent landscapes of cultural and touristic importance. In Southeast Asia karst is associated with high species-richness and endemism in plants and seen as priority areas for the conservation of biodiversity. There has been little research into the floras associated with karst in South America, most of which occurs in Brazil. We therefore sought to evaluate the importance of Brazilian karst with respect to its species-richness and endemism. We sought to do so using curated plant specimen data in the Botanical Information and Ecology Network (BIEN) dataset. We show that, except for Amazonia, the BIEN dataset is representative of the Brazilian flora with respect to the total number of species and overall patterns of species richness. We found that karst is under-sampled, as is the case for much of Brazil. We also found that whilst karst represent an important source of plant diversity for Brazil, including populations of approximately 1/3 of the Brazilian flora, it is not significantly more species-rich or richer in small-range and endemic species than surrounding landscapes. Similarly, whilst important for conservation, comprising populations of 26.5–37.4% of all Brazilian species evaluated as of conservation concern by International Union for Nature Conservation (IUCN), karst is no more so than the surrounding areas. Whilst experimental error, including map resolution and the precision and accuracy of point data may have under-estimated the species-richness of Brazilian karst, it likely represents an important biodiversity resource for Brazil and one that can play a valuable role in conservation. Our findings are in sharp contrast to those for Southeast Asia where karst represents a more important source of species-richness and endemism. We also show that although BIEN represents a comprehensive and curated source of point data, discrepancies in the application of names compared to current more comprehensive taxonomic backbones, can have profound impacts on estimates of species-richness, distribution ranges and estimates of endemism.

## Introduction

Karst occupies ca 20% of Earth’s dry ice-free land^1^. Karst areas are of great socioeconomic importance, as they supply water to up to 25% of the world’s population^1^, are associated with rural poverty^2,3^, and represent landscapes of cultural and touristic importance^4^. We define karst as landscapes that are underlain by soluble rock (e.g. limestone, dolomite and gypsum) in which there is appreciable water movement arising from a combination of high rock solubility and well-developed secondary (fracture) porosity^1,2^.

Karst landscapes include areas of exposed rock and areas overlain by soil. Karst includes heterogeneous features, some of which are rock-dominated (carbonate and non-carbonate outcrops, caves, and sink holes) and others which are not (dolines, underground water courses, and soils). Karst vegetation reflects this heterogeneity, sharing some general properties of all rocky habitats and some features which are exclusive to karst. Shared general properties include low availability of water, high insolation and exposure to wind, and flash floods^5^. Features which are exclusive to karst include the export of weathered material below ground in solution, as opposed to as solids or in suspension, and above ground, high levels of Ca, Mg and K and very slow rates of soil formation^6^. High concentrations of Ca, Mg and K, the absence of surface water and very slow rates of soil formation pose several challenges for colonising plants^7^ and have led in some places to the development of a specialised flora, often derived from rapid diversifications^8,9^. Combined with the high heterogeneity of microhabitats^6,10^ these features could be expected to result in a relatively high frequency of endemic species as has been documented in Southeast Asia, Mexico and the Greater Antilles^6,10–14^. In Southeast Asia karst areas have been referred to as ‘arks of diversity’^15^ and the limestone Yunnan-Guizhou plateau of southwest China, is recognised as a centre of plant diversity^16^ within which caves represent an important focus for species discovery^17,18^.

Studies documenting the plant diversity on karst are urgently required as karst vegetation is vulnerable due to the growing worldwide demand for limestone for cement production^19^, to rocky desertification caused by deforestation on karst^20^ and to its sensitivity to drought from climate change^3^. All of these considerations resulted in the risk to biodiversity and ecosystem services associated with karst being identified as a conservation issue of global importance^21^.

Within South America, karst landscapes represent 2% of the land area (370,809 km^2^)^22^, the majority of which are in Brazil, where it comprises 5–7% of the terrestrial surface and occurs in all phytogeographic domains^23^. Despite well-documented associations between karst and endemism elsewhere, and an awareness that rock outcrops contribute substantially to regional biodiversity^10^, there have been few attempts to evaluate the biodiversity value of karst in the Neotropics where it receives little recognition other than for its cave faunas. The vegetation of karst outcrops of Central and South America has been the subject of a small number of micro-ecological studies^12,24–28^, but there has been little analysis of macroecological or regional-scale patterns of richness and endemism on karst and low recognition within civil society of its biodiversity importance. For example, neither of two recent reviews of the vegetation of rocky outcrops in Brazil^29,30^ recognise karst/limestone or carbonate outcrops as a distinct class despite its hydrological properties.

Two barriers to quantifying the contribution of karst vegetation to regional species pools have been: (1) obtaining spatial information on the distribution of karst outcrops, and (2) obtaining spatial primary biodiversity data at a regional scale. The publication of a global spatial data set for karst by Centro Nacional de Pesquisa e Conservação de Cavernas (CECAV)^31^, and the publication of data on plant distribution, abundance and traits by BIEN^32^ represented an opportunity to overcome these barriers. Prompted by these resources, we planned to assemble primary plant distribution data associated with karst in the tropics and, in the process, evaluate the robustness of the BIEN dataset. As a geographical focus we selected Brazil as it represents the biggest karst resource for South America, and the BIEN dataset is most complete for the Americas^32^. The analysis of this dataset would also represent a first attempt at a macro-ecological analysis of karst vegetation in Brazil.

The objectives of this study were to: (1) estimate the plant species diversity on karst in Brazil, (2) estimate the species’ range sizes as a measure of the richness of endemic species on karst, and (3) to assess the contribution of the Brazilian karst flora to the flora of Brazil. This enabled us to address the following research questions: (1) Does karst vegetation make a substantial contribution to Brazil’s plant diversity? (2) Is karst vegetation an important source of species of conservation concern?

## Methods

We aimed to mitigate some of the known limitations of species occurrence data^33^ by interpreting the data and results using expert field and taxonomic knowledge. We also aimed to quantify the potential problems with the use of aggregated datasets, such as the misnaming of the primary data because of conflicting taxonomies, collection bias that might result in climatic or spatial distortions by extensively validating the taxonomic identity and geographical coordinates.

### Vascular plants dataset for Brazil

We selected BIEN as a source of vascular plant data for Brazil because it represented the most comprehensive source of species distribution data for the Americas that has been subject to data cleaning for both the taxonomic and georeference data^32^. In addition, BIEN includes all datasets from Flora do Brasil 2020 (FB2020)^34^, the Global Biodiversity Information Facility (GBIF)^35^ and Tropicos^36^.

Cleaning of the BIEN data was done using the package *speciesgeocodeR*^37^. The function “GeoClean” available from the package offers a number of different tests to clean datasets with geographic coordinates. Each function argument represents a different cleaning step (Table S1). The cleaned dataset contained over one and a half million records representing 298 families, 3,770 genera and 34,388 species of vascular plants. Record frequency for individual species ranged from one (6,722 species) to 4,659 (*Casearia sylvestris*) with an average of 44 records per species. 

We used the private version of the BIEN 3.4.5 dataset which was current at the time that we undertook our first analyses (data file received on 19.04.2018). Subsequently a new version (4.0) has been published. In order to verify that the update had no impact on our results we compared both versions with respect to the number of species names, records and the number of accepted names.

A significant challenge in generating lists of plant names is sourcing an authoritative taxonomic backbone. In order to aggregate data from different sources data portals require a standard reference or backbone. The major vascular plant portals, Tropicos^36^, BIEN, FB2020^34^ and Plants of the World Online (POWO)^38^ do not share a common names backbone, resulting in discrepancies in taxon delimitation. Tropicos^36^ has a backbone based on the Gray Herbarium index with the later integration of The Plant List^39^ and ongoing individual curatorial interventions that has evolved over several decades (Davidse, Pers. Comm.). Tropicos^36^ forms the basis of the Taxonomic Names Resolution Service^40^ used to standardize name application in BIEN. FB2020^34^ incorporates its own backbone, originally published as the Lista do Brasil having been custom-built from subsets of The International Plant Names Index (IPNI)^41^, the World Checklist of Vascular Plants (WC)^42^ and Tropicos^36^, with each name relevant to Brazil being assigned a taxonomic status by a family specialist. WC^42^, based on IPNI^41^ and ongoing review of taxonomic literature, is collated by specialist compilers and now almost complete, providing the taxonomic backbone for POWO^38^. Because a species check-list was outside the scope of the present study, analyses were carried out under the assumption that taxonomic errors were randomly distributed across the dataset. We did, however, calculate an estimate of that error against the taxonomic backbones of FB2020^34^ and WC^42^ for the whole dataset (Brazil), the study area and areas identified by karst in CECAV^31^.

A common source of incongruence between taxonomies is the resolution of synonymy. That is, where an entity considered a single taxon in one taxonomy is treated as several in another. Such discrepancies can lead to the over- or under-estimation of species richness, range-size(s) and endemism, as one population could be considered to be several populations of distinct taxa, or vice versa. Over- or under- estimates of these key biodiversity metrics can have significant real-world implications as the presence of endemic species is important in the recognition of areas that are important for plant diversity^43^ and range size estimates are among the main data used for assessing extinction risk applying IUCN criteria^44^. In order to evaluate the potential impact of synonymy in the BIEN dataset on our estimates of species number and range-size, we calculated the number of synonyms in WC^42^ compared to FB2020^34^. For the species in WC^42^ or FB2020^34^ with ten or more synonyms we looked at the effect of these differences in taxonomic circumscription on the number records recovered against each taxon name. The rationale for doing so was that the species for which BIEN accepted the largest number of synonyms were likely to have the biggest impact on estimates of biodiversity, endemism and range-size in our analysis.

### Karst dataset and the study area

We used a map of karst areas produced by CECAV^31^. In all spatial analyses, we used the South America Albers Equal Area Conic projection. For the purposes of the more detailed analysis the study area was limited to a bounding box within which all karst areas lie (Fig. S1). Given that nearly half of the georeferenced data from BIEN had an error of 5 km, we created a 5 km buffer zone around the polygon representing the extent of karst in Brazil (Fig. S2). Most of the further analyses were carried out for two karst extents, without a buffer zone (NBZ) and with a 5 km buffer zone (BZ5). The extents of karst used in our study comprised 3.7–6.3% of the land area of Brazil occurring in all of its biomes (Table 1). Using CECAV^31^, we defined the study area as a bounding box within which all karst areas lie, an area which represents ca 57% of the territory of Brazil (Table 1).

**Table 1:** Brazilian vascular plant species in the whole country, in the study area and in the two karst extents: without a buffer zone (NBZ) and with a 5 km buffer zone (BZ5). ^*^The number of endemic taxa in the whole country (Brazilian total) is from FB2020^34^.

Parameters of the subsets	Brazilian total (BT)	Study area		Karst extent			
				NBZ		BZ5	
		Value	% of BT	Value	% of BT	Value	% of BT
Area, km^2^	8,515,767	4,893,045	57.4	318,126	3.7	541,533	6.3
Number of distribution records	1,502,484	1,170,795	77.9	111,108	7.4	209,174	13.9
Number of taxa/species	34,388	28,818	83.8	9,592	27.9	13,174	38.3
Number of endemic taxa	18,639*	17,610	94.5	468	2.5	1,098	5.9
Maximum species richness in a 50 × 50 km grid cell	5,182	5,108	98.6	1,783	34.4	3,115	60.1

Because maps of carbonate outcrops or karst may not be comprehensive, due in part to the resolution of the maps often being lower than the dimensions of the outcrops, we validated our map using collectors’ notes associated with plant collections metadata stored in GBIF^35^, Herbário Virtual da Flora e dos fungos^45^, REFLORA^46^, CV Starr Virtual Herbarium^47^, Species Link^48^, and Tropicos^36^. We filtered collections from Brazil using the following keywords associated with karst, ‘calcario’, ‘calcário’, ‘calcaria’, ‘calcária’, ‘calcareo’, ‘calcáreo’, ‘calcarea’, ‘calcárea’, ‘calcareous’, ‘caliza’, ‘limestone’, ‘dolomito’, ‘dolomite’, ‘dolina’, ‘doline’, ‘carste’, ‘karst’, ‘carstica’, ‘cárstica’, ‘carstico’, ‘cárstico’, ‘cave’, ‘caverna’, and ‘gruta’. For those descriptors not exclusively associated with carbonate substrate we included records following further filtering. For keywords, ‘dolina’ and ‘doline’ records were checked manually to confirm that they were not associated with other substrates. In the case of ‘cave’, ‘caverna’, ‘gruta’, all of which can occur in other formations, e.g. campos rupestre, we retained only records that fell within a 1 km radius of other carbonate records. The geolocation of each record was then reviewed, and where necessary, corrected using http://splink.cria.org.br/geoloc. The taxon name for each record was reviewed and corrected against https://github.com/karstflora/CheckNamesBrazilianFlora2020. Records were then overlaid on the map of karst areas. In the case of records returned on the keywords ‘dolina’, ‘doline’ the geographical position of records was checked manually and localities not in recognised karst or limestone areas excluded, as such formations can occur in other rock types. In the case of records returned on the keywords ‘cave’, ‘caverna’, and ‘gruta’ the geographical position of records was checked manually and localities not within 1 km of recognised karst or limestone areas excluded, because such formations can occur in other rock types, such as sandstone.

### Species richness, weighted endemism, range size and conservation status

All analyses were performed in R version 3.4.3^49^. Species richness was measured as the total count of taxa within 50 × 50 km grid cells. This grid cell size is commonly used in regional biogeographic studies (e.g. Thuiller *et al*.^50^) and is also suitable for our data, because it provides a good balance between collating enough points for the purposes of the study and giving resolution appropriate for ecological interpretation of results. We calculated species richness for Brazil as a whole, for the study area, and for the two karst extents (NBZ and BZ5).

In order to ensure that differences in species-richness were not artefacts of sample intensity, we tested the strength of the relationship between the number of observations and species richness within NBZ and BZ5 by using a square root transformation of both datasets fitted to ordinary least squares (OLS) models^51^.

To determine the expected null distribution of species richness within the NBZ extent, we took 1,000 random draws of occurrence data from the NBZ species data. We then built species accumulation curves using “specaccum” function in R package *vegan*^52^ and 10 × 10 km grid cells as a unit area. It has been shown that species-area relationships (SAR) are scale dependent, however, variations in SAR parameters can only be observed when the difference in scale is large (e.g. communities vs evolutionary provinces)^53^. Although we did not test the effect of the increase in cell size on the SAR parameters, given the above consideration we adopted a five-fold increase in cell size (i.e. from 10 × 10 to 50 × 50 km grid cell) as suitable for the purposes of the current study. To estimate the steepness of the species-area curve and the expected number of species in a 10 × 10 km grid cell, we fitted the Arrhenius power relationship^54^ (S = kA^Z^, where S = number of species, A = area, and k and z are fitted parameters) to each of the curves using “fitspecaccum” function in *vegan*^52^. The distribution of the expected species richness in each of the 50 × 50 km grid cells was derived from the Arrhenius power relationship^54^ with parameters estimated as above and the area approximated by the number of the non-empty 10 × 10 km grid cells in each 50 × 50 km cell. We compared the observed and the expected species richness using quantiles of the null distribution. Species richness outliers were assigned categorically, with −1 for lower outlier (below 25^th^ quantile), 1 for non-outlier (between 25^th^ and 75^th^ quantiles), and 2 for upper outlier (above 75^th^ quantile) for each 50 × 50 km grid cell.

To test the hypothesis that species richness within the NBZ extent was not significantly different from that in the study area we randomly sampled the study area 1,000 times, each sample containing the same number of grid cells as the NBZ extent. We then performed a two-sided Student’s t-test^51^ on the species richness data contained in the random samples of the study area where the null hypothesis was that the sample mean (i.e. the mean species richness in the area of the same size as the NBZ extent) was not statistically different from the species richness of the NBZ extent. We then repeated the above steps using the BZ5 extent. We also built species accumulation curve for the study area using “specaccum” function in R package *vegan*^52^ and 50 × 50 km grid cells as a unit area. We then compared the resulting curve with the species richness values corresponding to the NBZ (9,592) and BZ5 (13,174) karst extents.

The definition of endemism has generated continuous discussion in the literature. Definitions based on an *a priori* cut-off point in terms of absolute or relative range size or restriction are somewhat arbitrary^55,56^ and increasingly superseded by methods that weight metrics of endemism with respect to taxon range sizes and/or species richness. Crisp *et al*.^55^ proposed a definition of ‘weighted endemism’ (WE), in which species richness is weighted by the inverse of the range size of each species, so that pools of species that occur over smaller ranges are given higher scores. Corrected weighted endemism (CWE) index obtained by dividing WE by the total count of species in the grid cell measures the proportion of endemics in that cell^55^. CWE highlights areas that have a high proportion of range-restricted species and so is valuable for prioritising areas for conservation. It does, however assume a linear relationship between the number of endemic species and species area which has been demonstrated not to be the case^57^. To avoid this effect, we used WE index estimated by weighting species richness in each cell by the inverse of the range size of each species, calculated as the number of 50 × 50 km grid cells occupied by that species. Calculations of WE were made using the self-contained R function developed by Guerin *et al*.^57^ with cell weights defined as “cell-based” (i.e. equivalent to “Area of Occupancy” as opposed to “Extent of Occupancy”). This approach is justified by the relatively large number of species restricted to a single grid cell (6,722). To determine the expected null distribution of endemic species for each observed value of species richness we ran randomisation tests as implemented by Guerin *et al*.^57^ with 1,000 replicates. Specifically, for each value of observed species richness, the expected null distribution of endemic species was determined by taking 2,000 random draws of that number of species from the overall pool; this null distribution was compared to the observed weighted endemism score to estimate statistical significance of the observed score being higher or lower than expected^57^. We calculated WE for the study area and for each of the two karst extents (NBZ and BZ5). As a result of limiting the extent of the analysis by karst areas, some non-karst species may appear to be karst endemics due to the fact that their main distribution range lies outside the karst area and only a small part of this range happens to be on karst. Thus, WE in the karst areas is likely to be overestimated. In our case, this approach is justified by a large number of small-range species often restricted to a single grid cell.

To estimate species range sizes within the two karst extents, NBZ and BZ5, and within the study area we used “lets.rangesize” function from the R package *letsR*^58^. This function estimated range size as a number of 50 × 50 km grid cells occupied by a species. Given the results of previous studies (e.g. Kreft^59^), we expected small-range species, defined as being restricted to a single grid cell, to be more frequent.

Because some of the species which on karst have small-ranges might have broader ranges elsewhere, we also calculated range size for all species within the study area. To test the hypothesis that the proportion of small-range species within the NBZ extent was no different from that across the study area, we randomly sampled the study area 1,000 times, each sample containing the same number of grid cells as the karst area within the NBZ extent. We then performed a two-sided Student’s t-test^51^ on the proportion of small-range species data contained in the random samples of the study area where the null hypothesis was that the sample mean (i.e. the mean proportion of small-range species within the area of the same size as the NBZ extent) was not statistically different from the proportion of small-range species in the NBZ extent. We then repeated the above steps using the BZ5 extent.

In order to evaluate the importance of karst with respect to the presence of threatened species we compared the species-list that we had generated for karst with the IUCN Red List^60^ of species whose extinction threat has been assessed.

With respect to land-use and conservation planning, the number of species endemic to an area is a more meaningful measure for comparing ecosystems than species number, especially with respect to vulnerability to environmental change^61^. In order to see how karst compares (with respect to the number of species endemic), to Brazil’s phytogeographic domains and to karst in Southeast Asia, we plotted the log of the number of endemic species against the log of surface area.

## Results

### Georeference and taxonomic bias

Data records with geo-reference and nomenclatural errors unresolvable by cleaning algorithms resulted in the loss of 3.9% of the records (Table S1). Using FB2020^34^ and WC^42^ as the reference for taxon names we calculated that 14.6–16.6% of the names applied to records in BIEN v3.4.5 are treated as synonyms in FB2020^34^ or WC^42^ (Table 2). For the subset of karst-associated records, the frequency of such names rose to 17.5–21.6% (Table S2). We extracted 1,170,795 records representing 28,818 species from BIEN v 3.4.5 for the study area, of which 111,108 (karst extent without a buffer zone, NBZ) and 209,174 (karst extent with a 5 km buffer zone, BZ5) were associated with karst (Table 1). In order to verify that our use of BIEN v3.4.5 rather than v.4.0 had no impact on our results we compared both versions with respect to the number of species names, records and the number of accepted names in agreement with WC^42^ and FB2020^34^ (Table 2). This showed that the discrepancy in names with respect to both WC^42^ and FB2020^34^ had increased, from 16.6 to 20.8% (WC) and 14.6% to 16.4% (FB2020).

**Table 2:** Comparison of the taxonomy of the Brazilian flora in BIEN to that used in the World Checklist of Vascular Plants (WC^42^) and the Flora of Brazil 2020 (FB2020^34^).

	BIEN v3.4.5 (sp = 34388)		BIEN v4 (sp = 52526)	
	WC (%)	FB2020 (%)	WC (%)	FB2020 (%)
Number of synonymous names (surplus names)	5708 (16.6)	5004 (14.6)	10914 (20.8)	8642 (16.4)
Number of shared names (in agreement)	26916 (78.3)	24797 (72.1)	36478 (69.4)	29962 (57.0)
Number of names not found	1732 (5.0)	4380 (12.7)	5063 (9.6)	13523 (25.7)

The projection of records sourced from collector notes over our karst map (Fig. S3) shows strong congruence between the map of karsts generated by CECAV^31^ and the meta data from plant collections.

### Species richness, weighted endemism and range size

**Figure 1:**
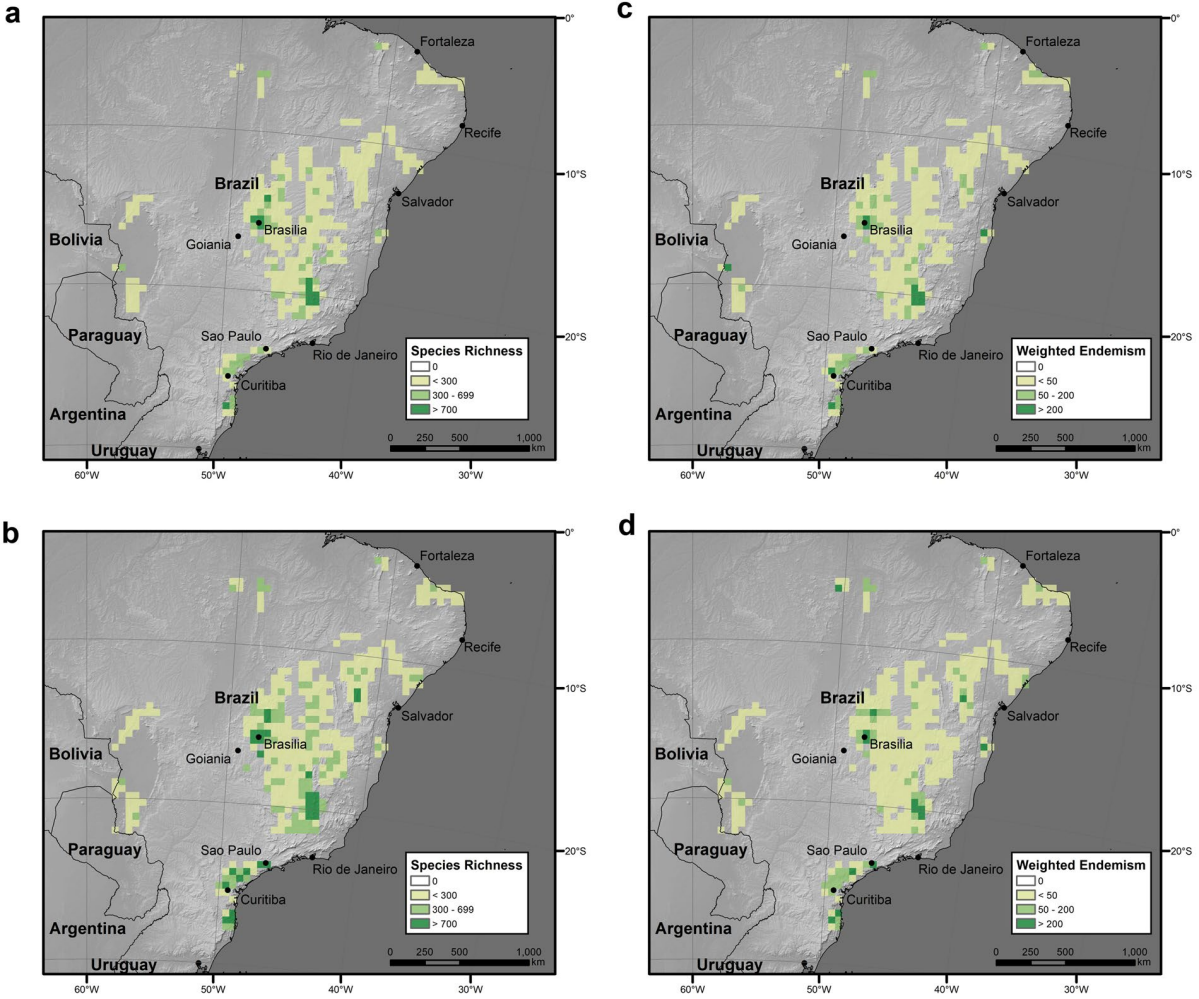
Species richness and weighted endemism of karst in Brazil. (**a**) Species richness in 50 × 50 km grid cells within the NBZ extent of karst. (**b**) Species richness in 50 × 50 km grid cells within the BZ5 extent of karst. (**c**) Weighted endemism in 50 × 50 km grid cells within the NBZ extent. (**d**) Weighted endemism in 50 × 50 km grid cells within the BZ5 extent. Map projection South America Albers Equal Area Conic. Software used to generate the maps: ESRI 2019. ArcGIS Desktop: Release 10.7. 1 Redlands, CA: Environmental Systems Research Institute; www.esri.com.

Species richness (the total number of recorded species in a given area) increased with the area size (Table 1), and the patterns of species richness within NBZ and BZ5 karst areas were largely similar (Fig. 1a,b) and recovered a strong positive association between the number of observations and species richness (Figs S5 and S6, Table S3).

The application of a 5 km buffer zone around the extent of karst increased the number of species records and of NBZ and BZ5 endemics recovered nearly two-fold, from 468 species in NBZ to 1,098 in BZ5. The increase in the total number of taxa was less dramatic, from 9,592 to 13,174 (Table 1). The number of karst species distribution records was in the range 7.4–13.9% of all the collection localities available for Brazil and represented 27.9–38.3% of all taxa (Table 1). For comparison, karst represents 3.7–6.3% of the total land area of Brazil (Table 1).

**Figure 2:**
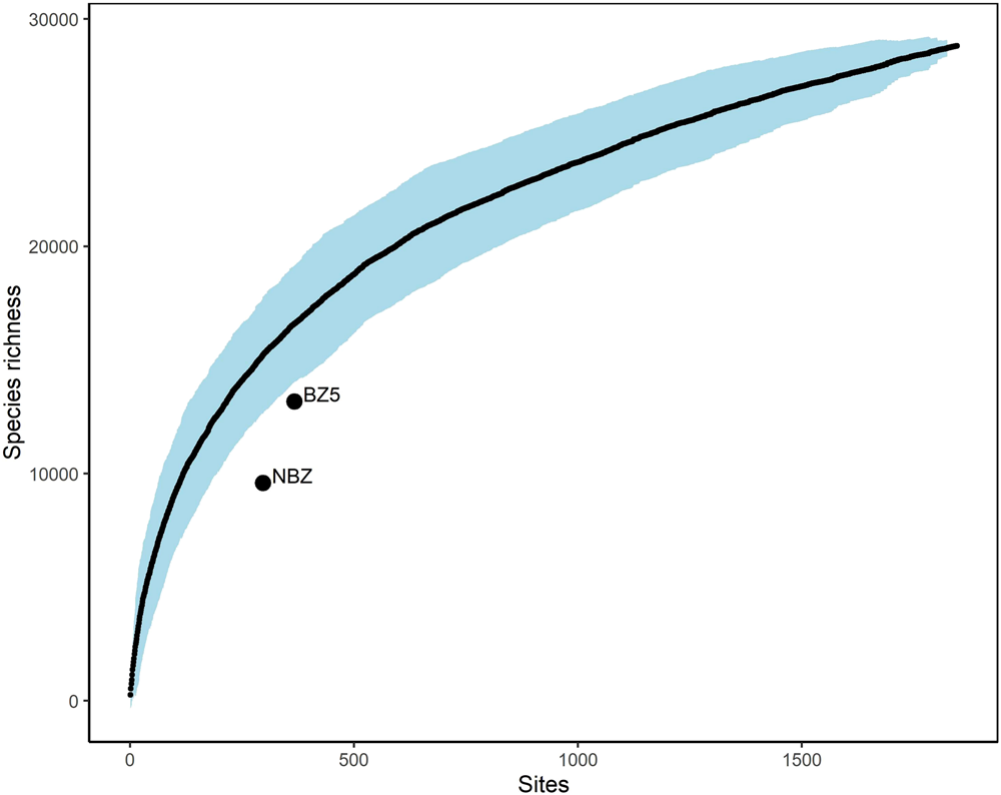
Species accumulation curve for the study area. Species richness values corresponding to the NBZ (9,592) and BZ5 (13,174) karst extents are outside the 95% confidence interval (shown in blue).

Comparison of the null distribution of species richness with the observed values suggests that the NBZ karst extent is under-sampled, because the majority of grid squares were identified as lower outliers (Fig. S7). The mean number of species in a 10 × 10 km grid cell estimated from 1,000 random draws within the NBZ extent was 297.21 (minimum = 114.81, maximum = 669.61, SD = 80.43), and the mean estimate of the slope of a species-area relationship was 0.48 (minimum = 0.36, maximum = 0.60, SD = 0.037). The Student’s t-test showed that observed values of NBZ and BZ5 species richness were significantly lower than the mean species richness across the study area (t = 134.36, df = 999, p-value < 0.001, and t = 82.365, df = 999, p-value < 0.001, Fig. S8). NBZ and BZ5 karst extents were outside the confidence interval of the species accumulation curve built for the study area (Fig. 2).

**Figure 3:**
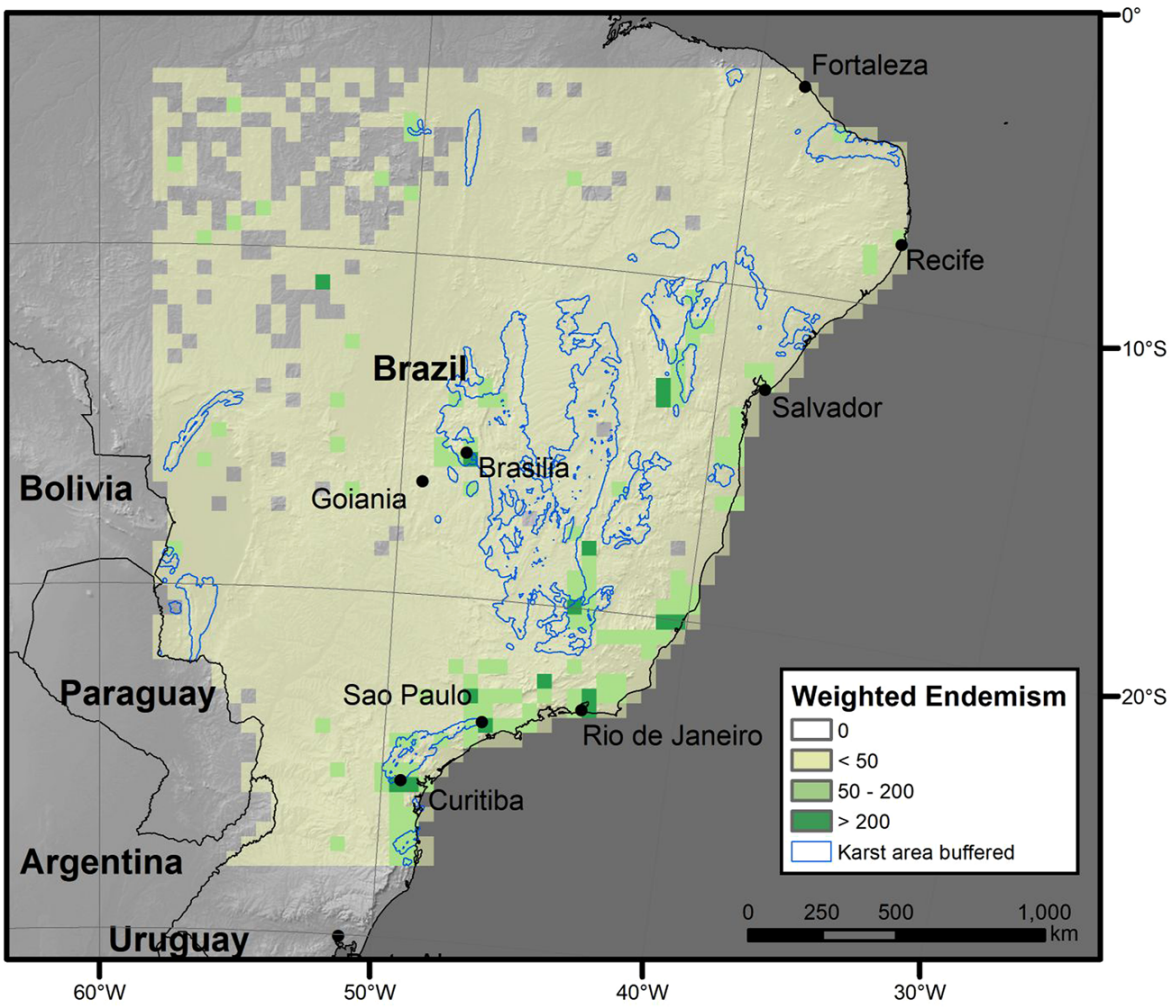
Weighted endemism in 50 × 50 km grid cells for the whole study area. Map projection South America Albers Equal Area Conic. Software used to generate the map: ESRI 2019. ArcGIS Desktop: Release 10.7. 1 Redlands, CA: Environmental Systems Research Institute; www.esri.com.

The patterns of weighted endemism were positively associated with those of species richness (Fig. S9) and largely similar between the two karst extents (Fig. 1c,d). The strongest positive outliers of weighted endemism were located in the more fragmented peripheral parts of the Brazilian karst, while the strongest negative outliers were clustered in the central portions of the karst areas (Figs S10 and S11). This pattern may be attributable to the fact that weighted endemism was calculated within the karst extent only. In order to explore this possibility we additionally calculated weighted endemism for the whole of the study area (Fig. 3). This recovered a pattern congruent (Fig. 3) to that for the karst area and so makes it unlikely that our findings for weighted endemism (Fig. 1c,d) are artefacts of having focussed on karst extent. Without ground-truthing it is impossible to tell whether these patterns are genuine or artefacts of sample error.

Range-size distribution of all vascular plant species calculated as the number of 50 × 50 km grid cells occupied by those species followed a typical “reversed J” pattern within all extents (Fig. S12), confirming the expectation that small-range species would be most abundant; and the number of small-range species (i.e. confined to a single grid cell) increased with sample area. In contrast the proportion of those species in the overall species pool was negatively related to the sample area, and within the study area, BZ5 and NBZ were 29.37%, 37.51% and 42.66% respectively. When a conservative measure of range size was applied to karst species, i.e. the range size of a species was estimated not within a karst extent, but within the study area, only 5.29% and 7.75% of the NBZ and BZ5 karst species respectively were confined to a single grid cell (Fig. S13).

There was a positive relationship between the size of an area and the share of small-range (i.e. confined to a single 50 × 50 km grid cell) species in the pool of species confined to this area (Fig. S14). A t-test confirmed that small-range species were underrepresented in the karst areas defined by the NBZ and BZ5 extents (t = 33.314, df = 999, p-value < 0.0001, and t = 21.12, df = 999, p-value < 0.0001, Fig. S15). Note that the distributions were bimodal (Fig. S15), and so whilst t-test is believed to be robust with respect to the violation of assumptions of normal distribution, the result may have been affected by the shape of the distribution.

### Conservation status

Depending on the definition of the karst extent, from 111 to 166 of the species associated with karst were classified as extinct (E), extinct in the wild (EW), critically endangered (CR), endangered (EN) or vulnerable (VU) according to IUCN criteria^44^. This represents 22.5–34.7% of all Brazilian species assessed as of conservation concern (Table 3), and 28.7–44.3% of the 384 species of conservation concern within the study area.

**Table 3:** Brazilian species of conservation concern according to IUCN Red List of Threatened Species^60^; EX: extinct; EW: extinct in the wild; CR: critically endangered, EN: endangered; VU: vulnerable; NT: near threatened; Conservation concern: all of the above categories combined; LC: least concern.

Categories	Brazil, 100%	Karst, NBZ		Karst, BZ5		Study area	
		Taxa	%	Taxa	%	Taxa	%
EX	5	0	0.0	0	0	5	100
EW	2	1	50.0	1	50	2	100
CR	55	6	11.0	15	27.3	45	81.8
EN	157	37	23.6	60	38.2	134	85.4
VU	271	67	24.7	90	33.2	198	73.1
NT	43	9	20.9	19	44.2	34	79.1
**Conservation concern**	**533**	**120**	**22.5**	**185**	**34.7**	**418**	**78.4**
LC	747	268	35.9	369	49.4	663	88.8
**Total**	**1280**	**388**	**30.3**	**554**	**43.3**	**1081**	**84.5**

**Figure 4:**
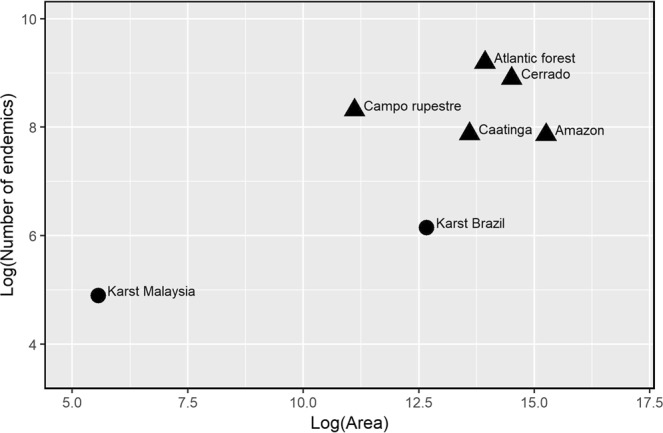
Number of endemic species in karst areas of Southeast Asia and Brazil, and in the main Brazil’s phytogeographic domains.

The plot of the log of the number of endemic species against the log of surface area (Fig. 4, Table S4) which provides a comparison of karst to Brazil’s phytogeographic domains and to karst in Southeast Asia, suggests that within Brazil, karst is a lower outlier.

### The BIEN dataset as a source of Brazilian vascular plant distribution data

BIEN currently holds data on over 34,000 taxa for Brazil which is comparable to that estimated for Brazil (33,161^62^ and 34,611^34^ species). The distribution of regional species richness is consistent with current understanding of the general richness patterns in Brazil (Fig. S4). Thus, the Atlantic Forest is confirmed as the most species rich Phytogeographic Domain^63^, while the Amazon Rainforest appears to be species-poor, likely as a result of insufficient sampling effort (Fig. S5). With respect to the taxonomy used in BIEN we found that 75.6% of the names in BIEN v4 used for the 1,502,484 Brazilian collections (Table 1) were in agreement with the WC^42^ classification, and that 71.3% were congruent with the taxonomy of FB2020^34^ (Table 2).

### Geological maps as an accurate representation of karst distribution

Our search of GBIF^35^, Herbário Virtual da Flora e dos fungos^45^, REFLORA^46^, CV Starr Virtual Herbarium^47^, Species Link^48^, and Tropicos^36^ recovered 13,970 records, which after filtering and checking were reduced to 3,811 species for which we have strong evidence from the label data that they were collected in karst. Whilst the distribution of these records coincided well with the CECAV^31^ karst distribution map (Fig. S3), it suggested that a number of karst outcrops remain undocumented. Undocumented outcrops were most common in south west Brazil towards the border with Bolivia and Paraguay.

## Discussion

### Contribution of karst vegetation to Brazil’s plant diversity

Brazil is a megadiverse country estimated to encompass 33,161^62^ to 34,611^34^ species of vascular plants. For the first time, we provide an estimate of the proportion of that flora associated with karst, which we suggest is 28% (NBZ) to 38% (BZ5) in an area representing 3.7 to 5.7% of the terrestrial extent of the country. Whilst this figure is high compared to other landscapes such as Amazonia^64^ it is relatively low compared to other rocky landscapes such as Campo Rupestre^65^ where ca 15% of the Brazilian flora is associated with an area <1% of the terrestrial cover.

Our results suggest that 4.8 to 8.3% of all karst species are endemic to karst (Table 1) and that the majority of species growing on karst can also be found in the surrounding non-karst areas (Table 1). This may suggest that the power of environmental conditions associated with karst, such as a surplus of Ca and K or frequency of drought, to select for species at a given locality, ‘environmental filtering’^66^, is low, or that the habitat has only recently been colonised. Low levels of NBZ- or BZ5-endemic species could also be an artefact of the resolution of the maps used to delimit karst^31^, i.e. the maps may not be detailed enough to distinguish between exposed karst and non-karst areas or they may include karst buried below deep soil deposits derived from non-karst deposits. Several studies, however, provide evidence that, at the regional scale, the Brazilian karst vegetation cannot be differentiated based on floristic composition. For example, within the Cerrado Domain, within which much karst is located, karst does not form a distinct phytogeographic domain^67^. Rather, the species composition of Brazilian karst falls within the phytogeographic regions in which it is located, *cerrado*, *caatinga*, *Atlantic forest*^68,69^. In *caatinga*, the largest and most diverse dry seasonal tropical forest biome in the Neotropical region, analyses of woody plant distributions did not identify vegetation on karst outcrops as a distinctive floristic grouping^70^. In the *Atlantic forest* phytogeographic region, one of 35 global biodiversity hotspots for conservation prioritization^71^, ordination analyses of the species-by-site matrix segregated several rock outcrop vegetation types, however none was uniquely associated with karst^72^. In summary, despite potential errors associated with map resolution and relatively unequal sample effort across the study area (Fig. S5) there has likely been sufficient sampling of karst to conclude that karst does not represent a distinct phytogeographic unit for Brazil. Karst could more usefully be considered as a putative subunit of currently recognized phytogeographic regions in reflection of its unique hydrogeology and vulnerability to mining and climate change. Therefore, whilst karst harbours a substantial proportion of the Brazilian flora in a small area, it does so no more than adjacent non-karst areas (Figs 2, 4 and S8). Our conclusion that karst is not as species-rich, or rich in species restricted to it, compared to surrounding areas is supported by anecdotal field observations by botanists who have extensive experience of collecting in karst in South America (Bolivia, John Wood, pers.comm; Brazil, Pablo Hendrigo, pers.comm). Our findings therefore suggest a different pattern of plant diversity associated with karst in Brazil compared to Southeast Asia, where karst is recognized as a hotspot for species diversity and endemism^10,15^.

### Is karst an important source of species of conservation value for Brazil?

Our results (Figs 3, S10–13 and S15) show that there is both an under-representation of species with small range-sizes in the NBZ karst area compared to the study area which also comprises additional biomes, and fewer endemic species compared to Brazil’s other phytogeographic domains. In summary, therefore, we can quantify the density of karst-endemic species, which is lower but not greatly dissimilar to that for *cerrado* and as such we would argue that it should be considered as a similar conservation priority. Currently, with the exception of the associated caves, karst receives no legislative protection or conservation actions. This is despite threats to karst from the mining of limestone for cement production and its innate vulnerability to changes in rainfall due to its peculiar hydrology. Combined with a lack of research into its associated pant diversity, these active threats make Brazilian karst similarly vulnerable to land-use change as *Caatinga*, another under-studied biome^73^.

Karst areas contain about one-quarter to one-third of the species assessed as of conservation concern in Brazil according to the IUCN Red List of Threatened Species^60^. However, the Red List is a non-random and biased sample^74^. In addition, IUCN extinction threat assessments for plants are generally based on measurements of areas of the species distribution (Extent of Occurrence, Area of Occupation)^75^; and our results demonstrate that species with small range-sizes are underrepresented in the karst area (Fig. 3, S10–13, and S15) suggesting that karst would likely be poorer in threatened species than neighbouring non-karst areas. This is in part supported by comparing percentages of species assessed as threatened (Table 3) between the study area, BZ5 and NBZ where the proportion of species evaluated classed as threatened drops from 38.6% (study area) to 33.3% (BZ5) and 30.9% (NBZ).

### Comparisons with Southeast Asia

The relatively low recognition of karst as a floristic assemblage of conservation value in Brazil is in sharp contrast to Southeast Asia where karst is widely recognized as having distinct floristic assemblages rich in karst-endemics and being of high conservation value^15^. This difference in perceptions of karst between Southeast Asia and Brazil may reflect the lower floristic importance of karst in Brazil, or it may reflect cultural differences and research intensity. A comparison of our results to those of Chin^76^ for Peninsular Malaysia suggests that Southeast Asia does indeed have a much higher proportion of karst endemics than Brazil, 11% versus 4.9% (Brazil, NBZ, also Fig. 4). It also suggests a far higher density of karst-endemic species with 134 species over a relatively small area (260 km^2^) for Peninsular Malaysia compared to 468 species over an area a thousand times larger (318,126 km^2^) for Brazil (Fig. S16). If Chin’s^76^ observations are representative of the remainder of Southeast Asia, we propose, that karst is indeed of lower floristic importance in Brazil compared to Southeast Asia.

Understanding why there is such a difference in the proportion and density of the respective floras endemic to karst will generate important insights into the accumulation of species diversity on karst and of the importance of this substrate to plant evolution worldwide. We hypothesise that these differences are the product of contrasting paleoclimate histories in these regions and their impact on the chemical reactions which drive karstification, a reaction dependent on temperature and water^1^. Much of the present geography and biotic composition in both regions was formed during the Neogene (the 20 million years that preceded the Pleistocene)^77^. In Southeast Asia there is evidence that species diversity on karst is a product of high rates of karstification^78^, whereby the arising of the East Asian monsoons 20–15 Ma accelerated the dissolution of carbonate creating new habitats for calciphiles and resulting in a peak in speciation rates.

*Cerrado*, within which most Brazilian karst is found, is a woody savanna that varies from open grasslands to forests^79^. Evidence suggests that *Cerrado* formed ca 10 Ma or later and so potentially is a much younger formation than those observed in Southeast Asia^80^. It has been hypothesised that *Cerrado* species composition and diversity reflects a frequent exchange between it and neighbouring tropical rain forest and seasonally dry tropical forest biomes^80^. At that time the Brazilin karst likely experienced decrease in precipitation and climate cooling following the Mid-Miocene climatic optimum^81^. During the Last Glacial Maximum (LGM), the climate in the Cerrado domain would have been both cooler and drier, and the process of karstificaton therefore slower^82^. Inter-tableland depressions and the highland slopes of central Brazil may have acted as refugia for the associated species but not for calciphiles^82^. If, therefore, the model of species accumulation on karst proposed by Kong *et al*.^78^ is applicable to all karst habitats, during the LGM rates of calciphile speciation would have been reduced, and extinction rates would have remained stable or increased as the karst became much drier and as they were excluded from the refugia available to non-calciphile *Cerrado* species. Thus, circumstantial evidence suggests that karst communities in Brazil are the product of dispersal from surrounding areas, rather than speciation of calciphiles specifically adapted to limestone substrates.

### Experimental error

#### Sample effort

Most of the karst area had from 100 to 1000 collections of vascular plants per 50 × 50 km grid cell and not under-sampled in comparison to the surrounding areas (Fig. S5). The fact that we observed a high degree of correlation between species richness and sampling effort (Fig. S6, Table S3) suggests that species-richness in species-poor sites was underestimated, possibly because collectors prioritise species-rich over species-poor areas. This is further supported by the fact that the few grid squares with the highest species-richness fell below the regression line (Fig. S6) suggesting that they had been relatively well sampled as opposed to the majority of grid squares. As has been demonstrated by Feeley^83^ this suggests that sample effort within much of the study area and including karst, has been insufficient to generate accurate numbers for species richness and composition.

Most of the karst area had fewer than 300 species per 50 × 50 km grid cell (Fig. S5), and the comparison of the null distribution of species richness with the observed values suggests that the NBZ karst extent has fewer species per 50 × 50 km grid cell than expected (Fig. S7). This suggests that the confidence interval of species-area is large due to a few sites being exceptionally species-rich (Fig. 2). As a result, most of the sites appear to be species-poor (observed richness) compared with the null distribution. This result is corroborated by a recent study that used SDMs of the *Cerrado* herb–shrub flora to generate the pattern of botanical richness^68^. Within the area dominated by karst outcrops, the estimated richness values were up to 2,155 species per 5 arc-min (~9.3 km at the equator) raster cell. Although the initial dataset of 9,862 accepted herb–shrub species names used by Amaral *et al*.^68^ was comparable in size to ours (Table 1), the resulting estimates of species richness per unit area obtained were much higher than those reported by us. This discrepancy indicates that the BIEN dataset, while representative of the total size of the karst flora, likely misrepresents patterns of botanical richness because it is a record of observed distributions based on low and unequal sampling intensity^83^ rather than modelled distributions^68^.

#### The BIEN dataset as a source of Brazilian vascular plant distribution data

The fact that the total number of species is so similar to other estimates of Brazil’s flora suggests that our estimates of absolute species-number from BIEN are accurate. In contrast, our results suggest that 17–22% of the names associated with data points in BIEN have names not accepted by WC^42^ and or FB2020^34^ (Table S2). This suggests that BIEN data may not be a consistent source of names. In one case, a single name, *Myrcia splendens* (Table S2), was treated as 43 (with respect to WC^42^) or 30 (with respect to FB2020^34^) taxa in BIEN, which if geographically clustered would have an impact on calculated range-sizes, species niche models and estimates of species-richness. Despite these levels of discrepancy in the application of names, the fact that the total estimates for Brazil is so close to published estimates^62,84^, this suggests that a number of species, similar to that for synonyms, are missing from BIEN. We traced the incongruence of BIEN names to the use of the Taxonomic Names Resolution Service^40^ whose taxonomy contains elements imported from the Plant List and/or Tropicos^36^, neither of which purport to be comprehensive classifications. In order to evaluate the impact of these taxonomic discrepancies on our analyses we compared the taxonomy of those records for species endemic to karst areas (Table S2) and found similar levels of taxonomic congruence between the total dataset for karst-endemic and study-area-endemic species, 72.9% congruent with WC^42^ for NBZ endemics and 72.5% congruence with FB2020^34^. This suggests a similar source of naming errors across all data partitions and so should not have impacted our comparisons between karst and non-karst areas. We repeated the comparison for the latest version of BIEN (V4) and found an increase in discrepancy in the application of names with respect to both WC^42^ and FB2020^34^ suggesting that this remains a source of potential error.

#### Maps of carbonate outcrop

Coordinate uncertainty and map resolution have a big impact on perception of the vascular plant diversity patterns as demonstrated by the nearly 30% increase in the number of species and a doubling of the number of “karst endemics” with the application of a 5 km buffer zone. In addition, maps of carbonate outcrops or karst may not be comprehensive as the geology of the World’s terrestrial surface is not mapped to a resolution that would include outcrops below a specific size, likely 25 km^2^. For this reason, we tested our map using an independent source of data on karst, collector notes from plant collections. The projection of records sourced in this way over our karst map (Fig. S3) shows strong congruence between the map of karsts generated by CECAV^31^ and the meta data from plant collections. It suggests that CECAV^31^ have not omitted any large karst massifs. It does, however, suggest that the extent of karst in southwest Brazil towards the border with Bolivia and Paraguay has been under-estimated by geologists and that a significant number of small outcrops remain unobserved, notably in Amazonia (Fig. S3). This would suggest that we may have under-estimated the diversity of plants associated with karst.

The bimodal frequency distribution of small-range species (Fig. S15) suggests that the karst projection may have included species from domains other than the intended object of study. Their inclusion in our species pull could therefore account for the bimodal distribution of species-ranges, whereby the second peak corresponds to species from another domain.

## Conclusions

Karsts represent a major provider of ecosystem services, including the maintenance of freshwater ecosystem integrity, recreation and tourism, which is under threat from mining and climate change. Their floras have been very poorly studied in South America compared to elsewhere in the tropics. Using the BIEN data and maps of carbonate extent we provide a first review of the karst flora of Brazil in which we demonstrate that karst has similar if slightly lower levels of species richness and small-range (endemic) species compared to the biomes in which karst outcrops are located. We find high overlap between karst floristic composition and that of their surrounding biomes and no evidence that karst represents a distinct floristic unit, but rather that its species composition is derived from surrounding biomes. This contrasts with Southeast Asia where karst is associated with exceptional levels of endemism. This suggests major differences in how karst floras assembles across the tropics. We believe that better documentation of karst floras in South America will not only better support the conservation of karst and its aquifers but also enable the formulation and testing of hypotheses of species colonisation and diversification on karst, shedding light on what appear to be major differences within the Tropics.

## Supplementary information

Supplementary information

## Data Availability

The datasets generated during and/or analysed during the current study are available from the corresponding author on reasonable request.

## References

[1] Ford, D. C. & Williams, P. W. *Karst Hydrogeology and Geomorphology*. (Wiley, 2007).

[2] Aley T (1990). The karst environment and rural poverty. Ozarks Watch.

[3] Wong T-C, Luo T, Zhang H, Li S, Chu W (2016). The Socio-Economic Transformation of Rocky Karst Areas: Case Study of Qianxinan Prefecture, Guizhou Province, China. Malaysian J. Chinese Stud..

[4] Williams, P. *World Heritage Caves & Karst*. (IUCN, 2008).

[5] Walter, H. *Die Vegetation der Erde. Band I. Die tropischen und subtropischen Zonen*. (Gustav Fischer Verlag, 1973).

[6] Pérez-García EA, Meave JA (2004). Heterogeneity of xerophytic vegetation of limestone outcrops in a tropical deciduous forest region in southern México. Plant Ecol..

[7] Hao Z, Kuang Y, Kang M (2015). Untangling the influence of phylogeny, soil and climate on leaf element concentrations in a biodiversity hotspot. Funct. Ecol..

[8] Chung K (2014). Phylogenetic analyses of Begonia sect. Coelocentrum and allied limestone species of China shed light on the evolution of Sino-Vietnamese karst flora. Bot. Stud..

[9] Fu L (2017). Cytology and sexuality of 11 species of Elatostema (Urticaceae) in limestone karsts suggests that apomixis is a recurring phenomenon. Nord. J. Bot..

[10] Zhu X, Shen Y, He B, Zhao Z (2017). Humus soil as a critical driver of flora conversion on karst rock outcrops. Sci. Rep..

[11] Adams, C. D. *Flowering plants of Jamaica*. (University of the West Indies, 1972).

[12] Brewer SW, Rejmanek M, Webb MAH, Fine PVA (2003). Relationships of phytogeography and diversity of tropical tree species with limestone topography in southern Belize. J. Biogeogr..

[13] León H, Sauget JS (1946). Flora de Cuba Volumen I. Contrib. Ocas. del Mus. Hist. Nat. del Col. ‘De La Salle’.

[14] Liogier, A. H. *Antillean studies. I, flora of Hispaniola. Part 1, Celastrales, Rhamnales, Malvales, Thymeleales, Violales*. (Moldenke, 1981).

[15] Clements R, Sodhi NS, Schilthuizen M, Ng PKL (2006). Limestone Karsts of Southeast Asia: Imperiled Arks of Biodiversity. Bioscience.

[16] Davis, S. D., Heywood, V. H. & Hamilton, A. C. *Centres of plant diversity. A guide and strategy for their conservation*. (IUCN Publications Unit, 1994).

[17] Cardoso P (2012). Diversity and community assembly patterns of epigean vs. troglobiont spiders in the Iberian Peninsula. Int. J. Speleol..

[18] Monro AK, Bystriakova N, Fu L, Wen F, Wei Y (2018). Discovery of a diverse cave flora in China. PLoS One.

[19] Whitten T (2012). Protecting biodiversity. Int. Cem. Rev..

[20] Jiang Z, Lian Y, Qin X (2018). Rocky Desertification in Southwest China: Impacts, Causes, and Restoration Earth-Science Reviews Rocky. Earth Sci. Rev..

[21] Sutherland WJ (2012). A horizon scan of global conservation issues for 2012. Trends Ecol. Evol..

[22] World Map of Carbonate Rock Outcrops v3.0. Available at: https://www.fos.auckland.ac.nz/our_research/karst/index.html/. (Accessed: 12th December 2018).

[23] Auler, A. & Farrant, A. R. A brief introduction to karst and caves in Brazil. In *Proceedings University of Bristol Spelaeological Society*, 187–200 (1996).

[24] Aukema JE, Carlo TA, Collazo JA (2007). Landscape assessment of tree communities in the northern karst region of Puerto Rico. Plant Ecol..

[25] Baden HM (2016). A botanical inventory of forest on karstic limestone and metamorphic substrate in the Chiquibul Forest, Belize, with focus on woody taxa. Edinburgh J. Bot..

[26] Felfili JM, Nascimento ART, Fagg CW, Meirelles EM (2007). Floristic composition and community structure of a seasonally deciduous forest on limestone outcrops in Central Brazil. Brazilian J. Bot..

[27] Pérez-García EA, Sevilha AC, Meave JA, Scariot A (2009). Floristic differentiation in limestone outcrops of southern Mexico and central Brazil: a beta diversity approach. Boletín la Soc. Botánica México.

[28] Trejo-Torres JC, Ackerman JD (2002). Composition Patterns of Caribbean Limestone Forests: Are Parsimony, Classification, and Ordination Analyses Congruent?. Biotropica.

[29] Scarano FR (2007). Rock outcrop vegetation in Brazil: a brief overview. Brazilian J. Bot..

[30] Silva J (2017). Panorama sobre a vegetação em afloramentos rochosos do Brasil. Oecologia Aust..

[31] CECAV/ICMBio. Mapa das Regiões Cársticas do Brasil. (2009). Available at: http://www.icmbio.gov.br/cecav/images/stories/projetos-e-atividades/regioes_carsticas/regioes_carsticas_BR_SAD69_2009.zip. (Accessed: 9th February 2018).

[32] Enquist BJ, Condit R, Peet RK, Schildhauer M, Thiers BM (2016). Cyberinfrastructure for an integrated botanical information network to investigate the ecological impacts of global climate change on plant biodiversity. PeerJ Prepr..

[33] Meyer C, Weigelt P, Kreft H (2016). Multidimensional biases, gaps and uncertainties in global plant occurrence information. Ecol. Lett..

[34] Jardim Botânico do Rio de Janeiro. Flora do Brasil 2020. Available at: http://floradobrasil.jbrj.gov.br/. (Accessed: 26th October 2018).

[35] GBIF. The Global Biodiversity Information Facility. *What is GBIF?* Available at: https://www.gbif.org/what-is-gbif. (Accessed: 14th November 2018).

[36] Missouri Botanical Garden. Tropicos. Available at: http://www.tropicos.org. (Accessed: 12th December 2018).

[37] Zizka, A. & Antonelli, A. speciesgeocodeR: An R package for linking species occurrences, user-defined regions and phylogenetic trees for biogeography, ecology and evolution. *bioRxiv*, 32755, 10.1101/032755 (2015).

[38] POWO - Plants of the World Online. Available at: http://www.plantsoftheworldonline.org. (Accessed: 6th December 2018).

[39] The Plant List. (2012). Available at: http://www.theplantlist.org. (Accessed: 4th December 2018).

[40] Boyle, B. *et al*. The taxonomic name resolution service: an online tool for automated standardization of plant names. **14**, 16 (2013). 10.1186/1471-2105-14-16 PMC3554605 23324024

[41] The International Plant Names Index. Available at: https://www.ipni.org/. (Accessed: 6th December 2018).

[42] World Checklist of Vascular Plants. Available at: http://wcsp.science.kew.org/. (Accessed: 12th December 2018).

[43] Darbyshire I, Anderson S, Asatryan A (2017). Important plant areas: revised selection criteria for a global approach to plant conservation. Biodivers Conserv.

[44] IUCN. *IUCN Red List Categories and Criteria: Version 3.1*. (IUCN Publications Unit, 2012).

[45] Herbário Virtual da Flora e dos fungos. Available at: http://inct.florabrasil.net/en. (Accessed: 6th December 2018).

[46] REFLORA. Available at: http://reflora.jbrj.gov.br/reflora/herbarioVirtual/ConsultaPublicoHVUC/ConsultaPublicoHVUC.do/. (Accessed: 6th December 2018).

[47] CV Starr Virtual Herbarium. Available at: http://sweetgum.nybg.org/science/vh. (Accessed: 6th December 2018).

[48] Species Link. Available at: http://inct.splink.org.br. (Accessed: 6th December 2018).

[49] R Core Team. A language and environment for statistical computing (2017).

[50] Thuiller W, Pollock LJ, Gueguen M, Münkemüller T (2015). From species distributions to meta-communities. Ecol. Lett..

[51] Crawley, M. J. *The R book*. (John Wiley & Sons, Ltd, 2013).

[52] Oksanen, J. *et al*. *Vegan: community ecology package. R package version 2.3-5*. (R Foundation, 2016).

[53] Drakare S, Lennon JJ, Hillebrand H (2006). The imprint of the geographical, evolutionary and ecological context on species – area relationships. Ecol. Lett..

[54] Arrhenius Olof (1921). Species and Area. The Journal of Ecology.

[55] Crisp MD, Laffan S, Linder HP, Monro A (2001). Endemism in the Australian flora. J. Biogeogr..

[56] Laffan SW, Crisp MD (2003). Assessing endemism at multiple spatial scales, with an example from the Australian vascular flora. J. Biogeogr..

[57] Guerin GR, Ruokolainen L, Lowe AJ (2015). A georeferenced implementation of weighted endemism. Methods Ecol. Evol..

[58] Vilela Bruno, Villalobos Fabricio (2015). letsR: a new R package for data handling and analysis in macroecology. Methods in Ecology and Evolution.

[59] Kreft H, Sommer JH, Barthlott W (2006). The significance of geographic range size for spatial diversity patterns in Neotropical palms. Ecography (Cop.)..

[60] IUCN. The IUCN Red List of Threatened Species. Version 2018-2. Available at: http://www.iucnredlist.org. (Accessed: 14th November 2018).

[61] Kier G (2009). A global assessment of endemism and species richness across island and mainland regions. PNAS.

[62] Ulloa CU (2017). An integrated assessment of the vascular plant species of the Americas. Science (80-.)..

[63] Zappi DC (2015). Growing knowledge: an overview of Seed Plant diversity in Brazil. Rodriguésia.

[64] Cardoso D (2017). Amazon plant diversity revealed by a taxonomically verified species list. Proc. Natl. Acad. Sci. USA.

[65] Silveira FAO, Negreiros D, Barbosa NPU, Buisson E, Carmo FF (2016). Ecology and evolution of plant diversity in the endangered campo rupestre: a neglected conservation priority. Plant Soil.

[66] Kraft NJB (2015). Community assembly, coexistence and the environmental filtering metaphor. Funct. Ecol..

[67] Arruda DM, Fernandes-Filho EI, Solar RRC, Schaefer CEGR (2017). Combining climatic and soil properties better predicts covers of Brazilian biomes. Sci. Nat..

[68] Amaral AG, Munhoz CBR, Walter BMT, Jesus A-G, Raes N (2017). Richness pattern and phytogeography of the Cerrado herb – shrub flora and implications for conservation. J. Veg. Sci..

[69] Ratter JA, Bridgewater S, Ribeiro JF (2003). Analysis of the floristic composition of the Brazilian cerrado vegetation III: Comparison of the woody vegetation of 376 areas. Edinburgh J. Bot..

[70] Silva AC, Souza AF (2018). Aridity drives plant biogeographical sub regions in the Caatinga, the largest tropical dry forest and woodland block in South America. PLoS One.

[71] Myers N, Mittermeier RA, Mittermeier CG, da Fonseca GAB, Kent J (2000). Biodiversity hotspots for conservation priorities. Nature.

[72] Neves DM (2017). Dissecting a biodiversity hotspot: The importance of environmentally marginal habitats in the Atlantic Forest Domain of South America. Divers. Distrib..

[73] Moro MF, Nic Lughadha E, de Araújo FS, Martins FR (2016). A Phytogeographical Metaanalysis of the Semiarid Caatinga Domain in Brazil. Bot. Rev..

[74] Brummitt N (2015). The Sampled Red List Index for Plants, phase II: ground-truthing specimen-based conservation assessments. Philos. Trans. R. Soc. London B Biol. Sci..

[75] Collen B (2016). Clarifying misconceptions of extinction risk assessment with the IUCN Red List. Biol. Lett..

[76] Chin S (1977). The limestone hill flora of Malaya: Part I. Gard. Bull. Singapore.

[77] Hoorn C (2010). Amazonia Through Time: Andean Uplift, Climate Change, Landscape Evolution, and Biodiversity. Science (80-.)..

[78] Kong H (2017). Both temperature fluctuations and East Asian monsoons have driven plant diversification in the karst ecosystems from southern China. Molectular Ecol..

[79] Ribeiro, J. F. & Walter, B. M. T. As principais fitofisionomias do bioma Cerrado. In *Cerrado: ambiente e flora* (eds Sano, S. M. & Almeida, S. P.) 289–556 (EMBRAPA-CPAC, 2008).

[80] Simon MF (2009). Recent assembly of the Cerrado, a neotropical plant diversity hotspot, by *in situ* evolution of adaptations to fire. PNAS.

[81] Antonelli A, Sanmartín I (2011). Why are there so many plant species in the Neotropics?. Taxon.

[82] Bueno ML (2017). Effects of Quaternary climatic fluctuations on the distribution of Neotropical savanna tree species. Ecography (Cop.)..

[83] Feeley K (2015). Are We Filling the Data Void? An Assessment of the Amount and Extent of Plant Collection Records and Census Data Available for Tropical South America. PLoS One.

[84] Martins E, Martinelli G, Loyola R (2018). Brazilian efforts towards achieving a comprehensive extinction risk assessment for its known flora. Rodriguésia.

