# Supplementary material for: A Preliminary Evaluation of The Karst Flora of Brazil Using Collections Data
Source: Sci Rep. 2019 Nov 19;9:17037. doi: 10.1038/s41598-019-53104-6 (PMC6863846; doi:10.1038/s41598-019-53104-6)
Supplement: Supplementary file 1 — Supplementary information [file 41598_2019_53104_MOESM1_ESM.pdf]

Supplementary material for the article:

A PRELIMINARY EVALUATION OF THE KARST FLORA OF BRAZIL USING COLLECTIONS DATA

by Nadia Bystriakova*, Pablo Hendrigo Alves De Melo, Justin Moat, Eimear Nic Lughadha, Alexandre K. Monro

* Corresponding author: n.bystriakova@nhm.ac.uk; Core Research Laboratories, The Natural History Museum, London, SW7 5BD, UK

TABLES

Table S1. Cleaning steps (using R package *speciesgeocodeR*) and their results. Note that some coordinates were non-valid according to both tests 3 and 6; therefore numbers don't add up.

Test	Number of records FALSE	TRUE
1. Isna: checks for missing values in the coordinates	0	1584637
2. Isnumeric: checks for non-numeric values in the coordinates	0	1584637
3. Coordinatevalidity: checks for non-valid coordinates (XCOOR > 180 and < -180; YCOOR >90 and <-90)	16815	1567822
4. Haszero: checks for coordinates that are exactly zero	118	1584519
5. Zerozero: checks for coordinates that are within the rectangle around 0/0	35	1584602
6. Equallong: checks for rows where XCOOR = YCOOR	16370	1568267
**Summary of steps 1 to 6 (simple clean): removed/retained**	**17039**	**1567598**
7. Countrycentroid: checks if the coordinate fall within a rectangle with the length of one side 1.0 degree, around the centroid of the country (X=14.235; Y=51.9253).	1307	1566291
8. Country.check: checks if the coordinates fall within the country borders (NAs included in FALSE)	63807	1503791
**Summary of steps 7 to 8 (advanced clean): removed/retained**	**65114**	**1502484**

Table S2. Comparison of the taxonomy of the endemic Brazilian flora in BIEN to that used in the World Checklist of Vascular Plants (WC) and the Flora of Brazil 2020 (FB2020). Extent of karst: NBZ - without a buffer zone, BZ5 - with a 5 km buffer zone; % in brackets refers to the total number of species within each extent.

	Endemics of karst				Endemics of the study area (17609 species)	
	NBZ (468 species)		BZ5 (1098 species)			
	WC (%)	FB2020 (%)	WC (%)	FB2020 (%)	WC (%)	FB2020 (%)
Number of synonyms names (surplus names)	101 (21.6)	82 (17.5)	214 (19.5)	190 (17.3)	3680 (20.9)	3304 (18.8)
Number of shared names (in agreement)	341 (72.9)	281 (60.0)	810 (73.8)	655 (60.0)	12760 (72.5)	11261 (63.9)
Number of names not found	25 (5.3)	100 (21.4)	73 (6.6)	245 (22.3)	1150 (6.5)	2921 (16.6)
Number of unresolved names	1 (0.2)	5 (1)	1 (0.1)	8 (0.7)	20 (0.1)	124 (0.7)

Table S3. Characteristics of the OLS models describing the relationships between the number of observations and species richness in 50 x 50 km grid cells: NBZ – karst extent without a buffer zone; BZ5 – karst extent with a 5 km buffer zone.

Karst extent	R squared	Adjusted R squared	F statistics	Degrees of Freedom	p-value
NBZ	0.919	0.919	3380	295	< 0.001
BZ5	0.908	0.907	3585	365	< 0.001

Table S4. Number of endemic species in karst areas of Southeast Asia and Brazil, and in the main Brazil's phytogeographic domains.

Phytogeographic domain	Area, km^2	Number of endemic species
Amazon	4,198,273	2596
Atlantic forest	1,120,000	9835
Cerrado	2,000,000	7339
Caatinga	800,000	2637
Campo rupestre	66,450	4082
Karst Brazil	318,126	468
Karst Malaysia	260	134

Table S5. Number of BIEN synonyms and number of BIEN occurrences with the share of records attributed to synonyms (%) in brackets, of species in the World Checklist of Vascular Plants (WC) or Flora of Brazil (FB2020) with ten or more synonyms in BIEN.

Accepted names	FB2020		WC	
	No. of synonyms recognized in BIEN	No. of point records (% of records for accepted species)	No. of synonyms recognized in BIEN	No. of point records (% of records for accepted species)
Myrcia splendens (Sw.) DC.	43	5459 (34.5)	30	5135 (30.3)
Myrcia guianensis (Aubl.) DC.	37	3852 (14.8)	31	3728 (12.0)
Guatteria australis A.St.-Hil.	18	1568 (2.7)	18	1568 (2.7)
Myrcia tomentosa (Aubl.) DC	15	2382 (3.9)	17	2467 (7.3)
Lippia origanoides Kunth	13	302 (73.8)	13	302 (73.8)
Eugenia punicifolia (Kunth) DC.	13	2927 (0.8)	11	2923 (0.7)
Myrcia venulosa DC.	12	720 (3.1)	8	714 (2.2)
Guatteria punctata (Aubl.) R.A.Howard	11	393 (36.1)	11	393 (36.1)
Actinostemon klotzschii (Didr.) Pax	10	500 (29.6)	4	414 (15)

FIGURES

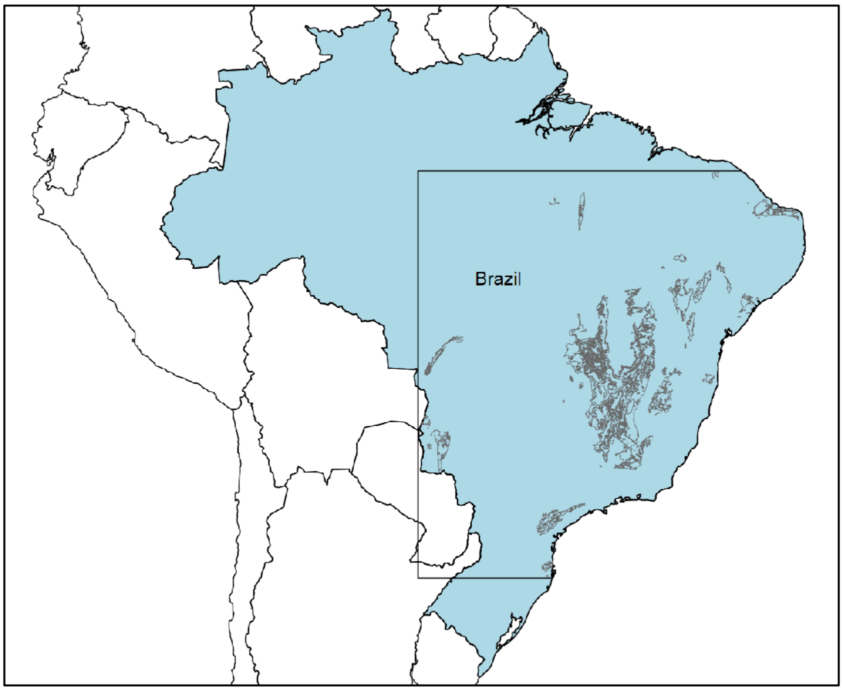

Fig. S1. The map of Brazil with the extent of the study area shown as a bounding box and the outline of the karst areas. Map projection South America Albers Equal Area Conic. The study area is limited by a bounding box within which all karst areas lie (xmin : 180000; xmax : 2742514; ymin : 441500; ymax : 3140000); although the extent of the bounding box covers parts of Argentina, Bolivia and Paraguay, only data relevant to Brazil have been used for the analysis.

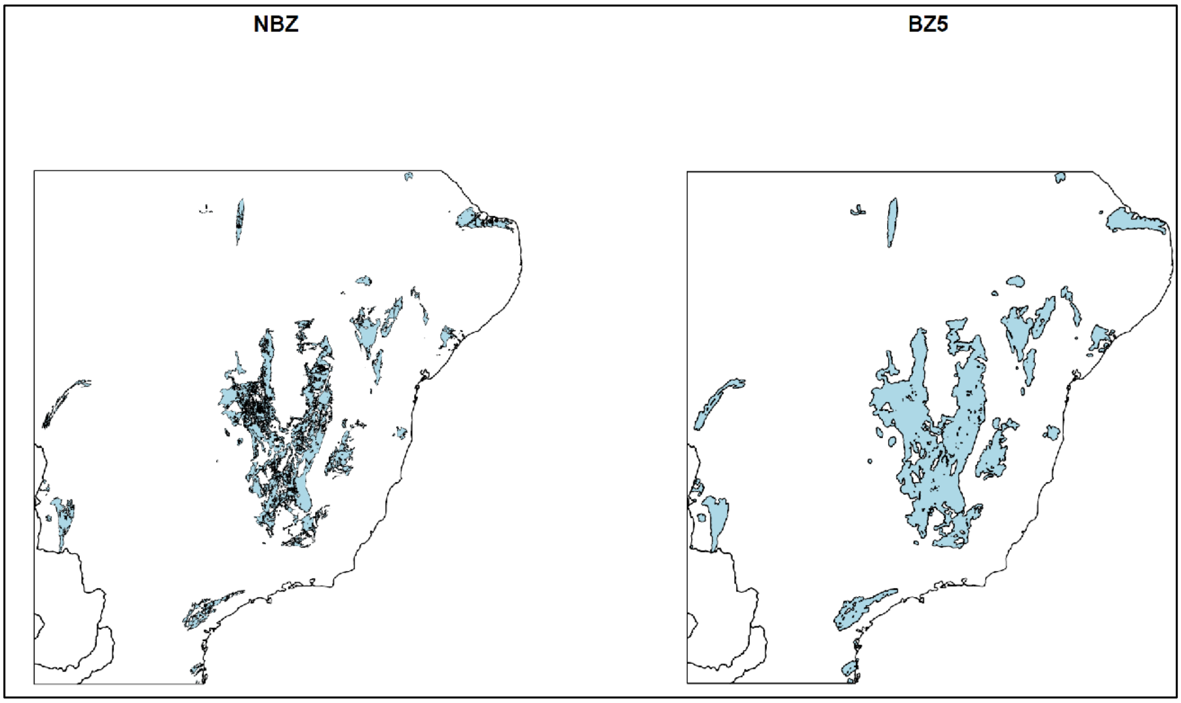

Fig. S2. Extent of the Brazilian karst used in the analysis: NBZ - without a buffer zone; BZ5 - with a 5 km buffer zone. Map projection South America Albers Equal Area Conic.

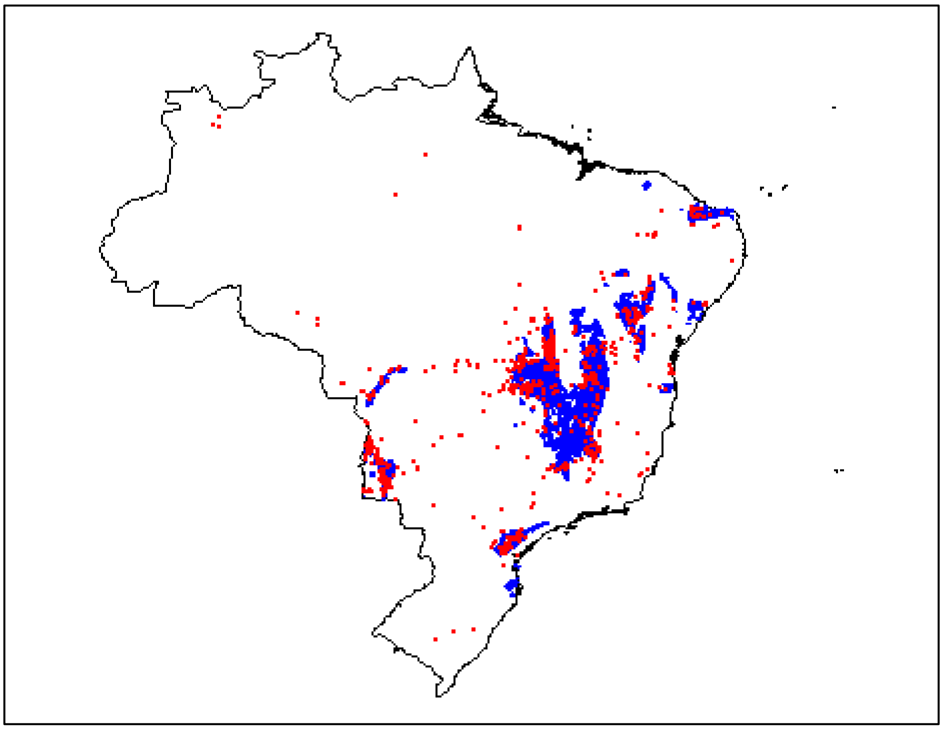

Fig. S3. Extent of the Brazilian karst used in the analysis (blue, NBZ) with localities from specimen records whose collector notes state that the locality was karst, limestone or carbonate (See Methods for wording). Map projection South America Albers Equal Area Conic.

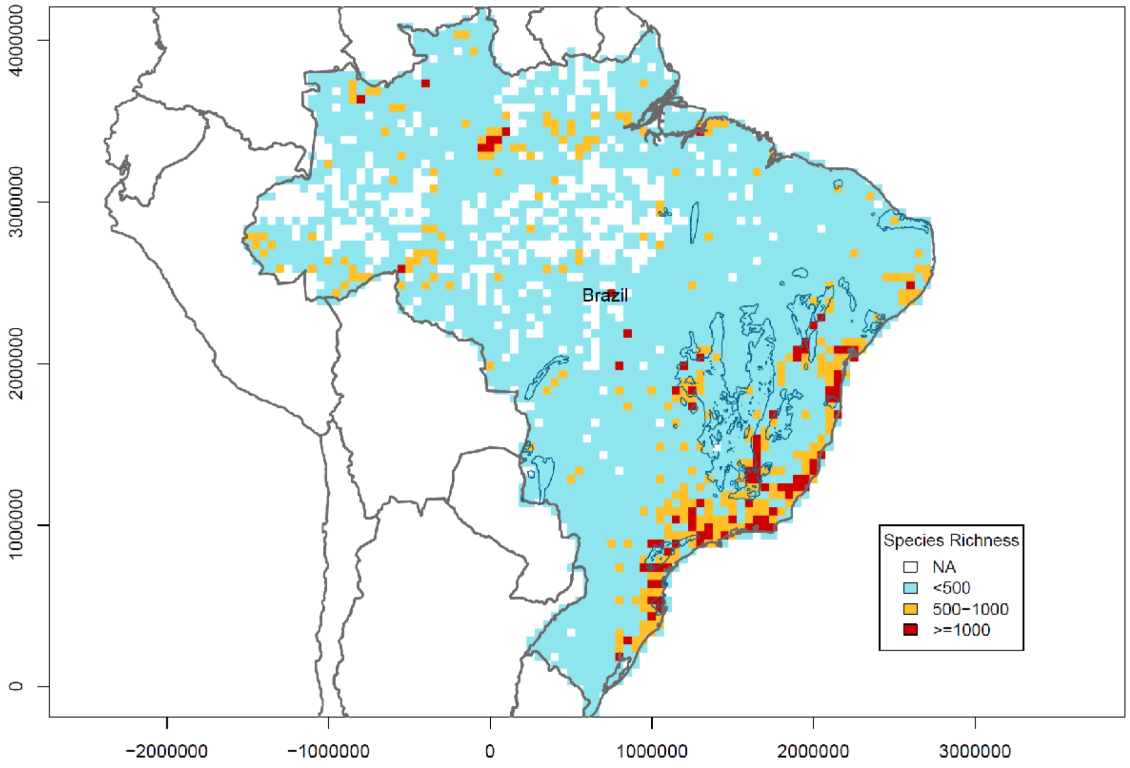

Fig. S4. Species richness map of Brazil. Grid cells are 50x50 km in size; map projection South America Albers Equal Area Conic. The extent of BZ5 is shown as an outline.

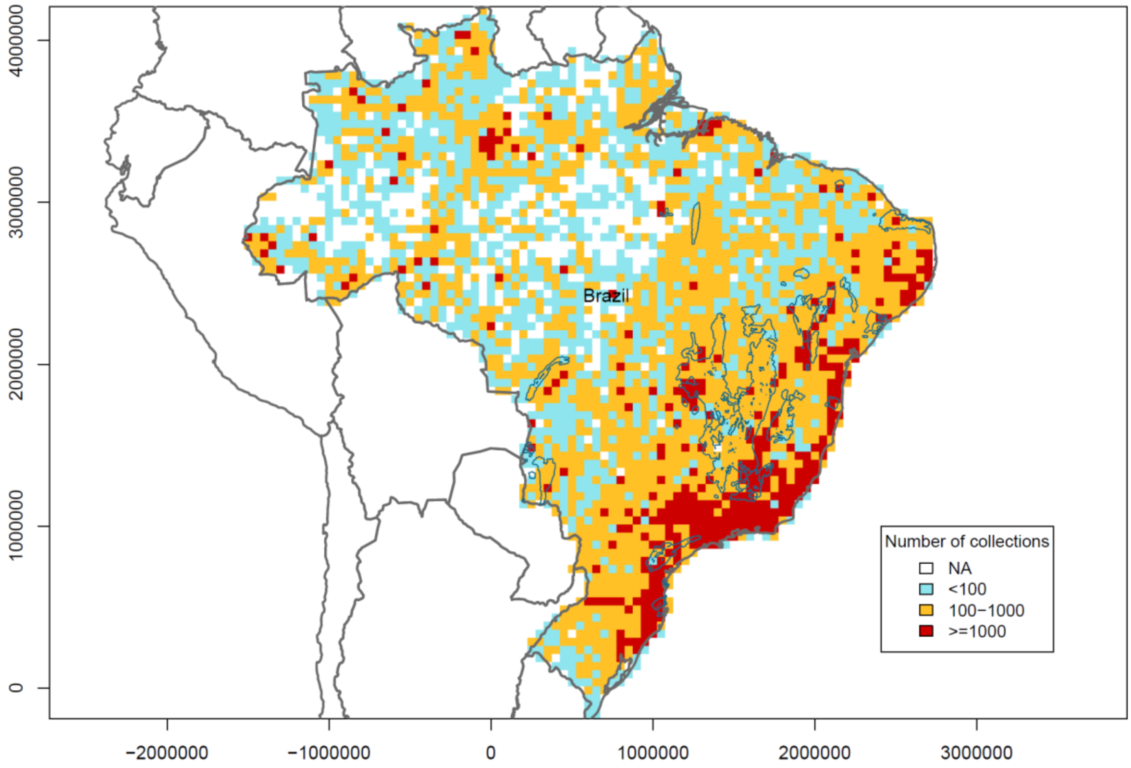

Fig. S5. Sampling intensity map of Brazil. Grid cells are 50x50 km in size; map projection South America Albers Equal Area Conic. The extent of BZ5 is shown as an outline.

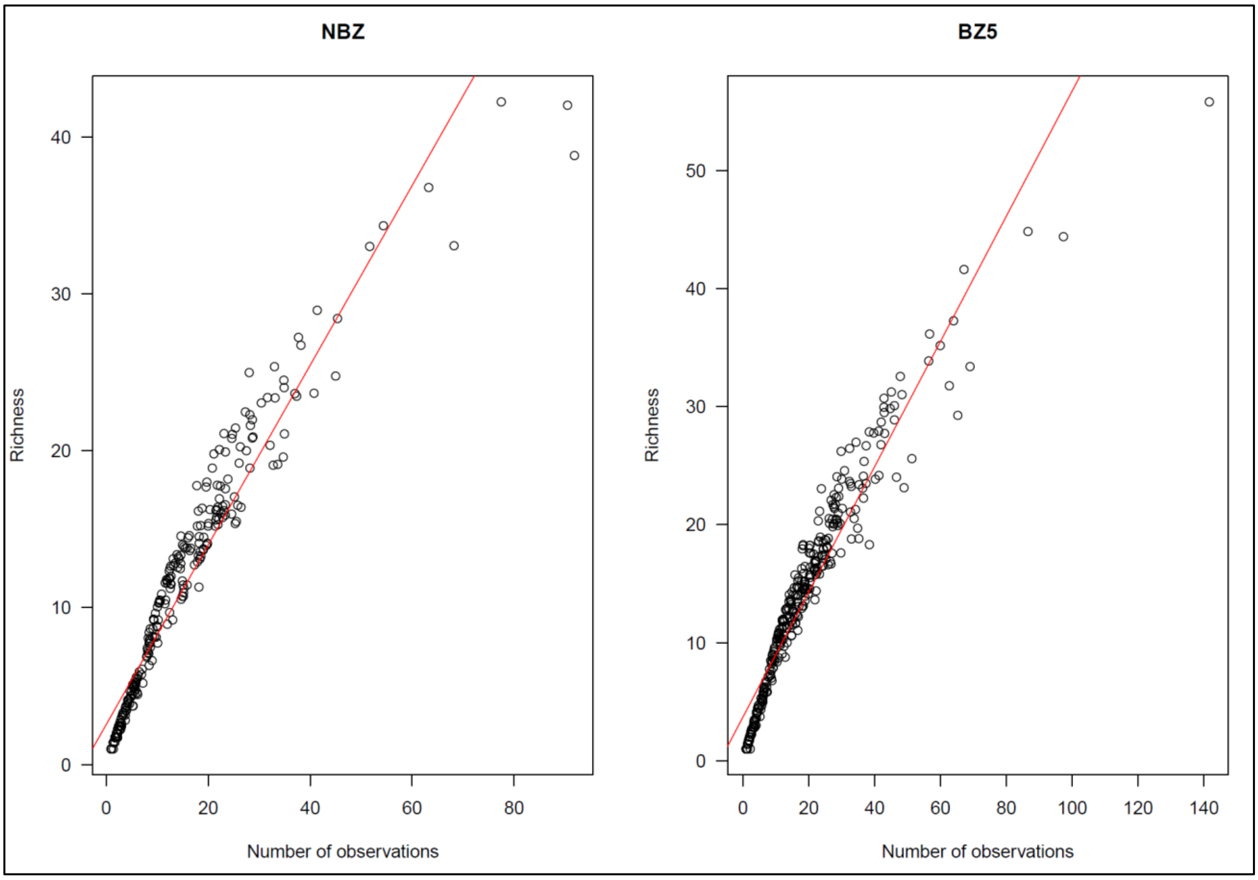

Fig. S6. Relationship between the number of observations and species richness in 50 x 50 km grid cells: NBZ – karst extent without a buffer zone; BZ5 – karst extent with a 5 km buffer zone.

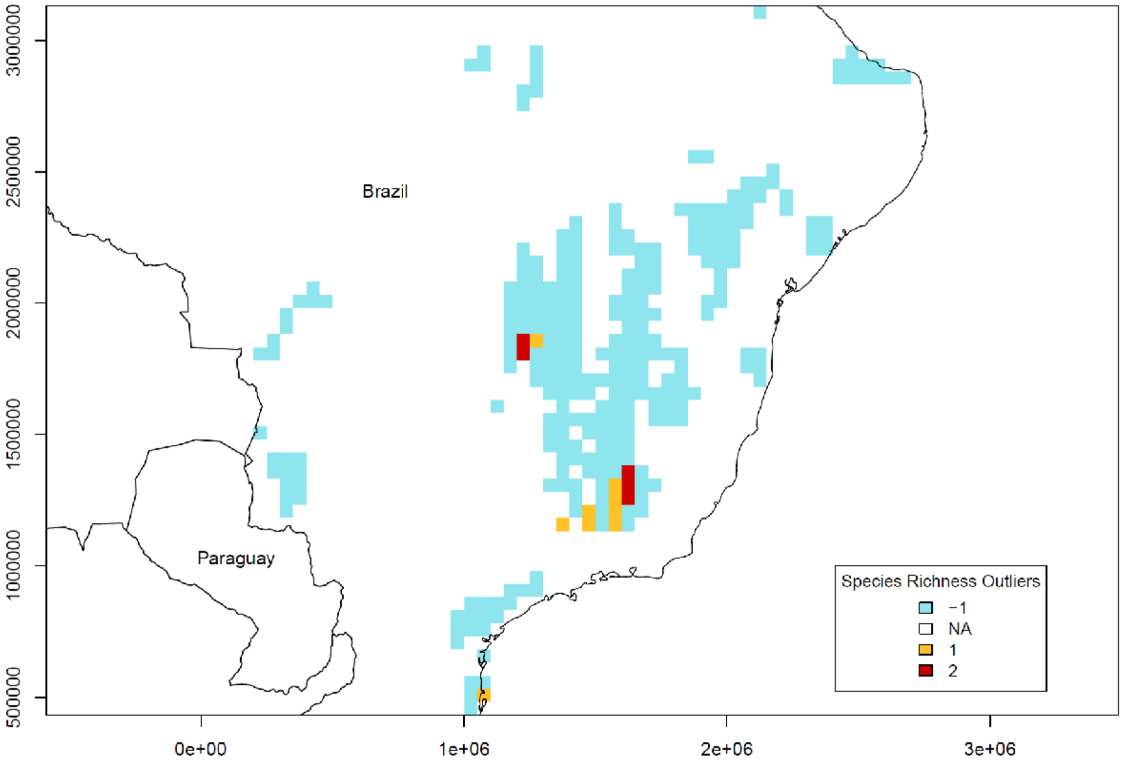

Fig. S7. Species richness outliers in 50x50 km grid cells within the NBZ extent: (-1) lower outlier (below 25th quantile); (0) no data; (1) non-outlier (between 25th and 75th quantiles); (1) upper outlier (above 75th quantile). Map projection South America Albers Equal Area Conic.

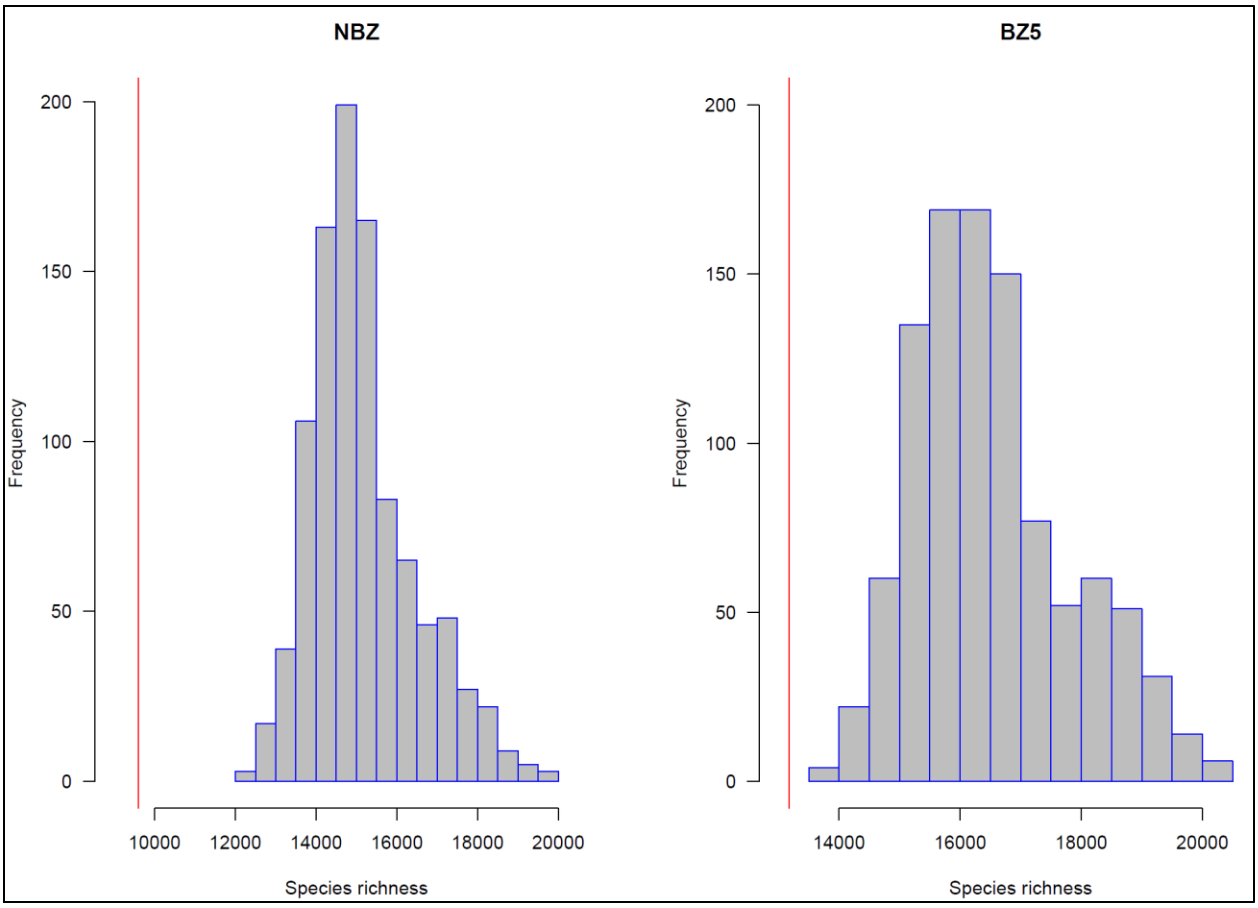

Fig. S8. Frequency distributions of species richness in the areas of the same size as the NBZ and BZ5 extents sampled randomly across the study area 1,000 times each. Red lines correspond to the observed species richness in NBZ (9,592) and BZ5 (13,174).

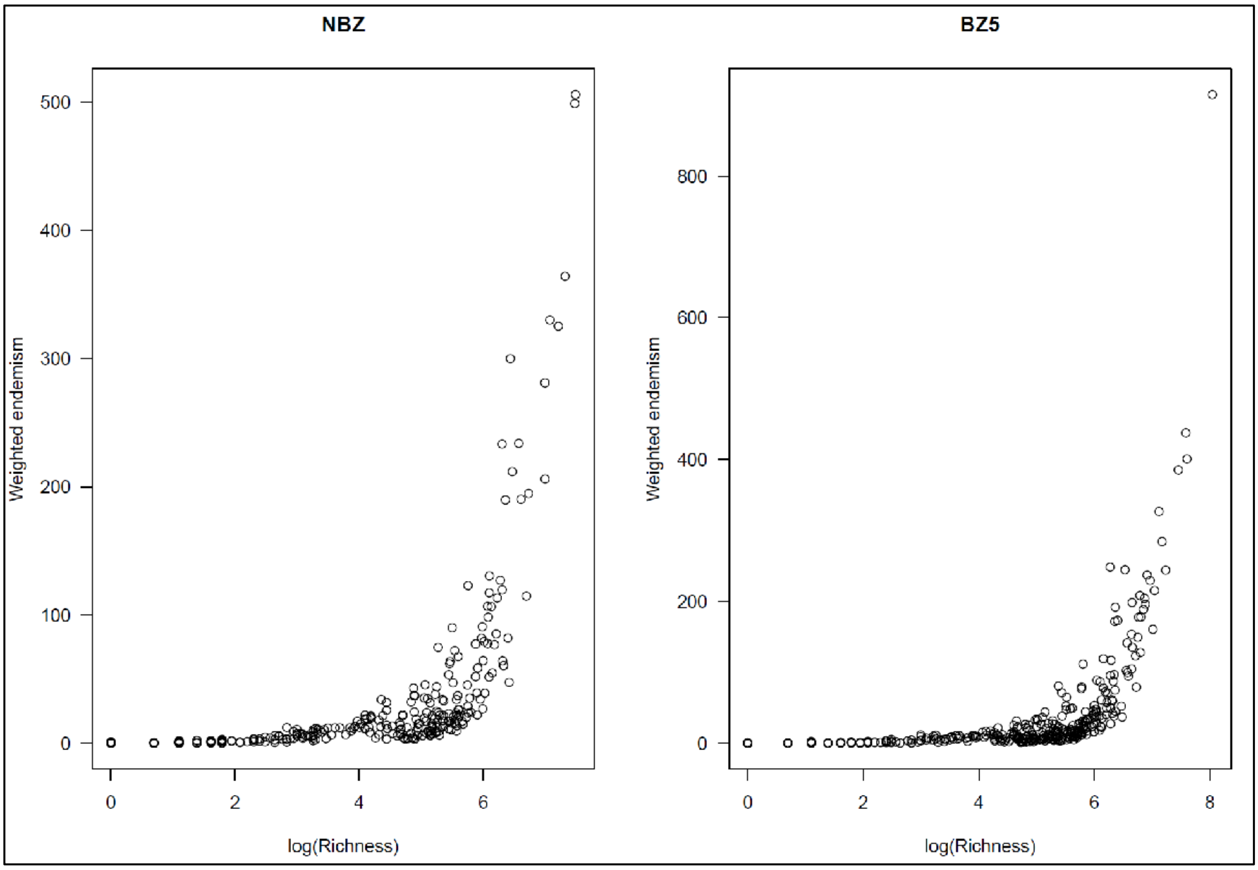

Fig. S9. Relationship between the number of species and weighted endemism in 50 x 50 km grid cells: NBZ – karst extent without a buffer zone; BZ5 – karst extent with a 5 km buffer zone.

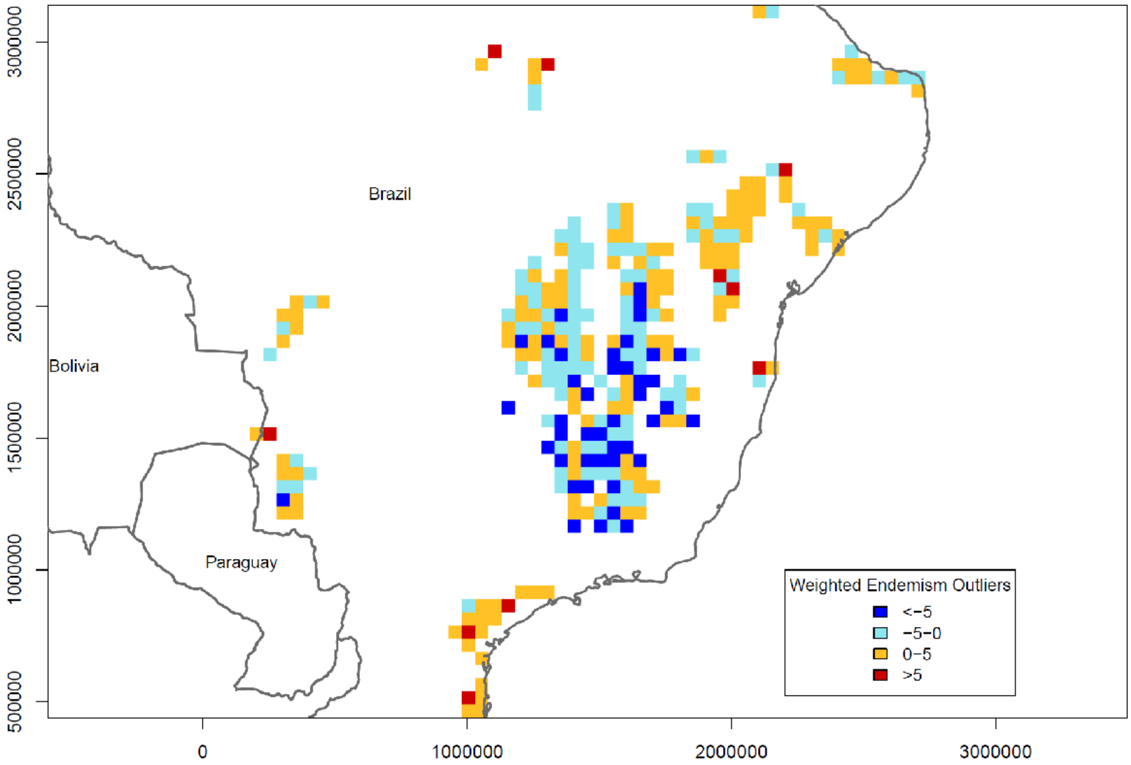

Fig. S10. Weighted endemism outliers within the NBZ (karst extent without a buffer zone). Scores for each grid cell indicate by which factor of the interquartile range observed endemism differs from the median / 50% quantile weighted endemism scores in ca 50 x 50 km grid cells. Warm colours (orange and red) indicate outliers with weighted endemism scores higher than expected, and the scores lower than expected shown in blue and light blue.

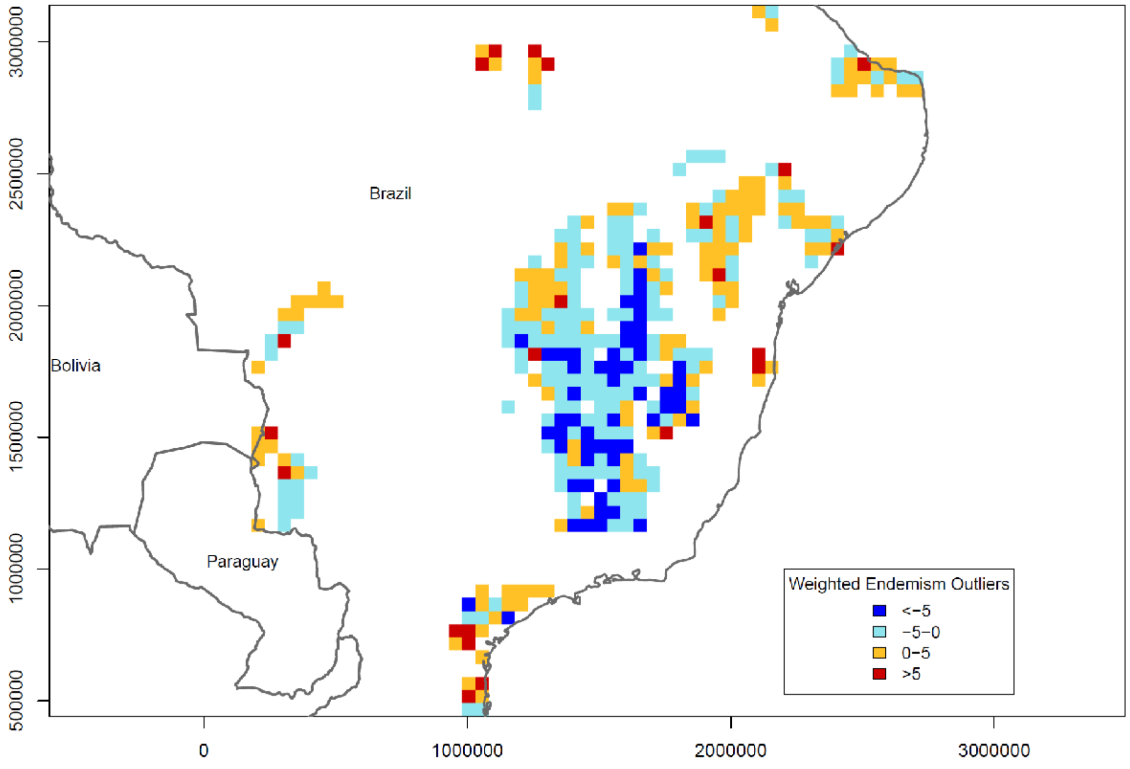

Fig. S11. Weighted endemism outliers within the BZ5 (karst extent with a 5 km buffer zone). Scores for each grid cell indicate by which factor of the interquartile range observed endemism differs from the median / 50% quantile weighted endemism scores in ca 50 x 50 km grid cells. Warm colours (orange and red) indicate outliers with weighted endemism scores higher than expected, and the scores lower than expected shown in blue and light blue.

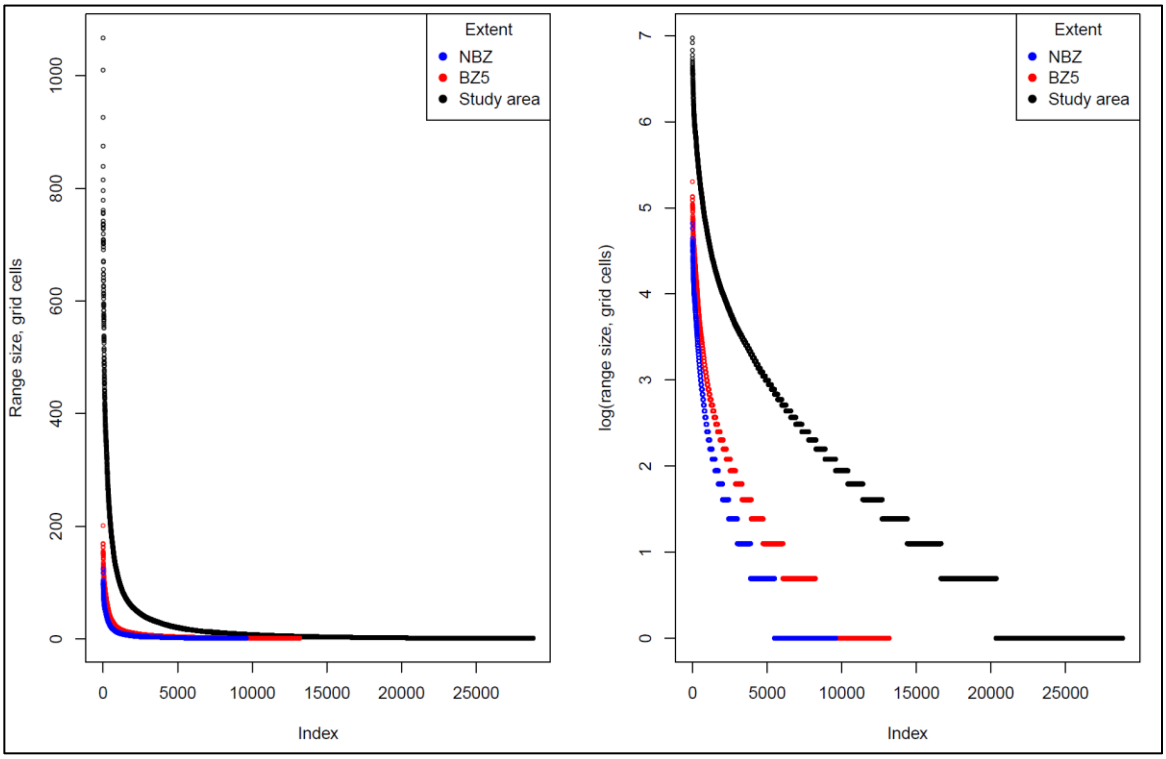

Fig. S12. Range size distribution as the number of grid cells (left) and the same number on logarithmic scale (right) occupied by vascular plant species within the study area and within the karst extents, NBZ and BZ5. The proportion of small range species (i.e. confined to a single grid cell) within the study area, BZ5 and NBZ was 29.37, 37.51 and 42.66 per cent correspondingly.

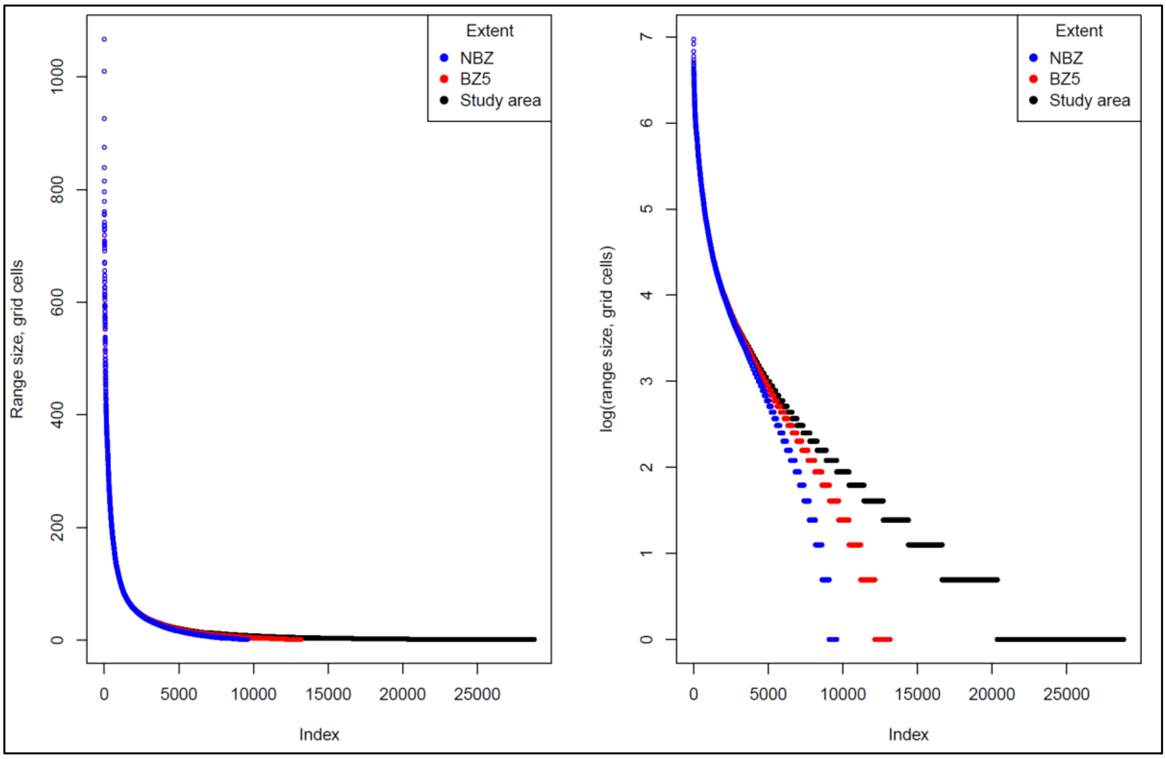

Fig. S13. Range size distribution as the number of grid cells (left) and the same number on logarithmic scale (right) occupied by vascular plant species within the study area. A conservative measure of range size was applied to karst species, i.e. range size of a species was estimated within the study area.

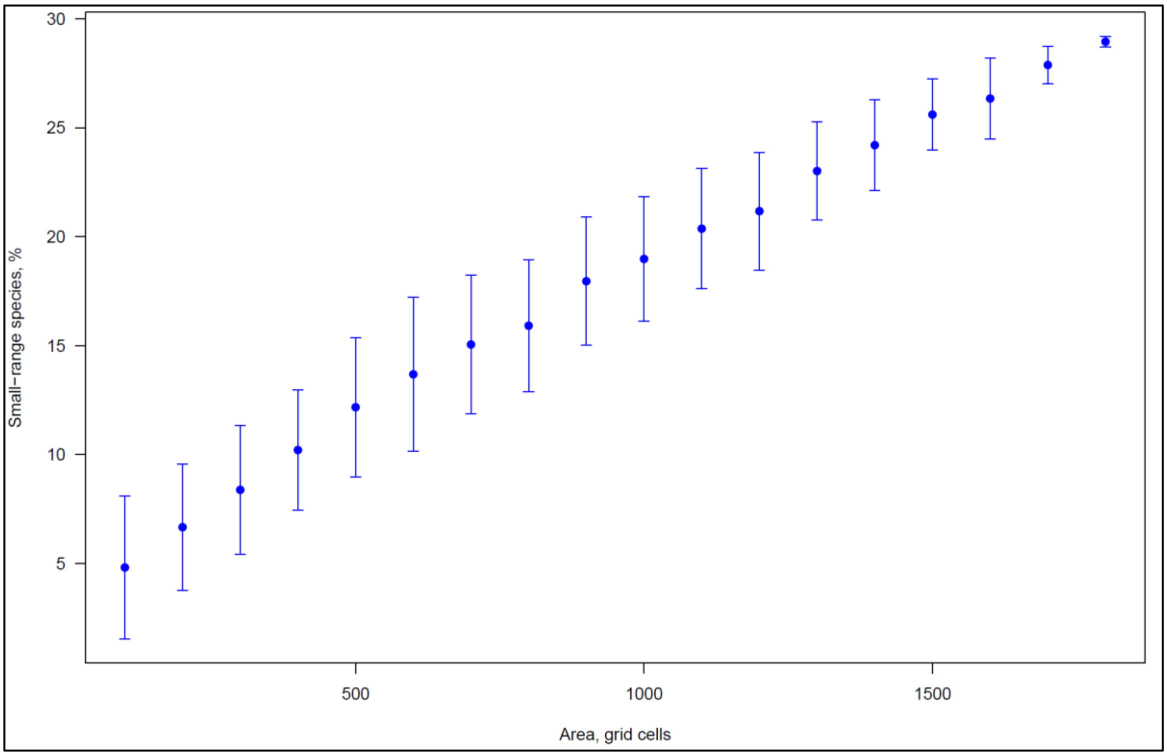

Fig. S14. Relationship between the area size and the share of small-range (i.e. confined to a single 50x50 km grid cell) species in the overall species pool. The mean values (dots) and error bars (two standard deviations of the mean) are calculated for 100 random draws without replacement of a number of grid cells corresponding to each area size.

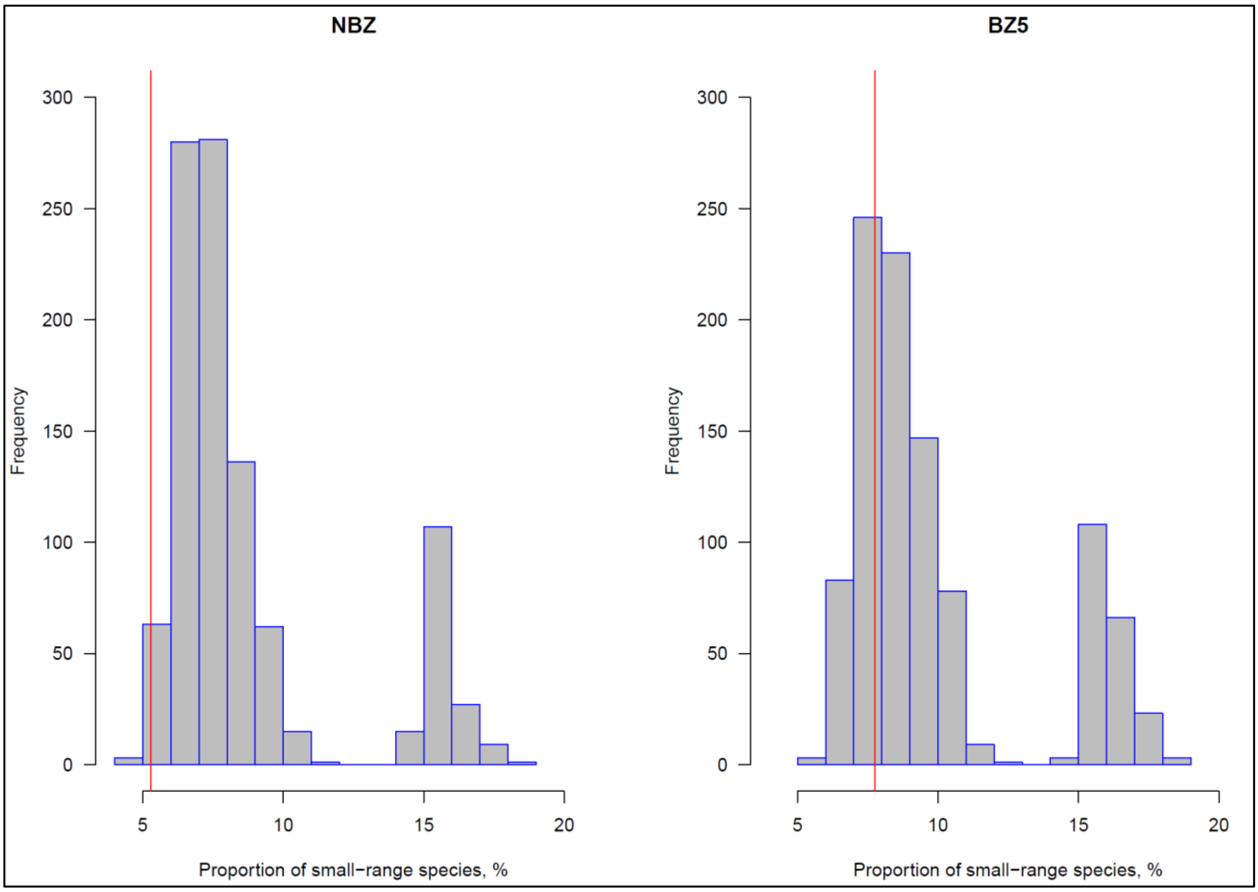

Fig. S15. Frequency distributions of the proportions of small-range species in the areas of the same size as the NBZ and BZ5 extents sampled randomly across the study area 1000 times each. Vertical red lines correspond to the proportions of small-range species in the NBZ (5.29%) and BZ5 (7.75%).

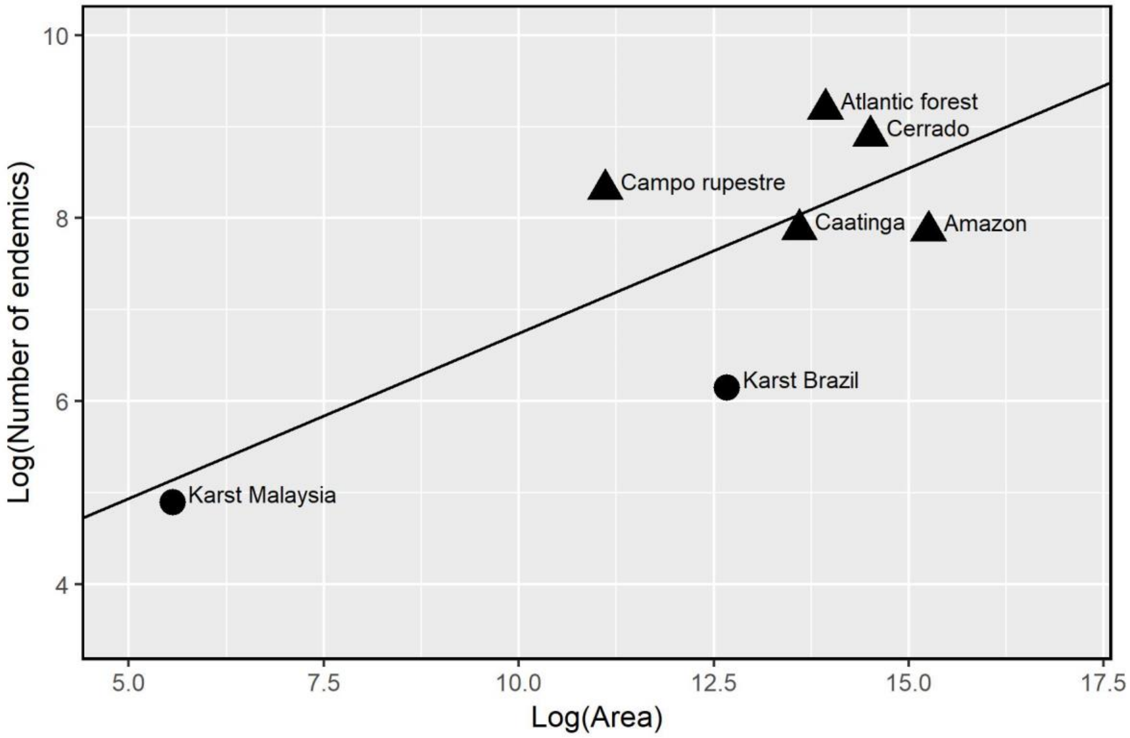

Fig. S16. Number of endemic species in karst areas of Southeast Asia and Brazil, and in the main Brazil's phytogeographic domains. The regression equation for the fitted line has a form: log(Number of endemics) = 3.13 + 0.36*log(Area), R-squared: 0.5915, F-statistic: 7.241, p-value: 0.04325. With the expected 2189 endemics as opposed to the observed 468, Karst Brazil falls well below the regression line, while Karst Malaysia with the expected 169 endemics as opposed to the observed 134 is very close to the line. Note that the parameters of the regression equation have been calculated for the dataset in which South East Asian karst is underrepresented.
